# Remediation of sandy loam soil contaminated with copper by washing assisted with ultrasound

**DOI:** 10.1016/j.ultsonch.2026.107893

**Published:** 2026-05-17

**Authors:** Rita Salameh, Antoine Leybros, Stéphanie Szenknect, Rachel Pflieger

**Affiliations:** a ICSM, Univ Montpellier, CEA, CNRS, ENSCM, Bagnols-sur- Cèze 30207, France; b CEA, DES, ISEC, DMRC, Univ Montpellier, Marcoule, France

**Keywords:** Soil remediation, Ultrasound, Heavy metals, Copper

## Abstract

Amongst heavy metal pollutants bound to soil constituents, copper is one of the most widely used, due to industrial and agricultural applications. This study investigated the combination of leaching with biosourced reagents with 45 or 358 kHz ultrasonic (US) irradiation. A soil was artificially contaminated with Cu and decontamination was performed on this unseparated soil and on two of its granulometric fractions: fine loam and fine sand. Mechanisms of dissolution/diffusion were modelled using parabolic equation. Silent washing of the unseparated soil with 0.2 M citric acid led to desorption of 61.9 % of the Cu after 1 h, similarly to 0.1 M HCl leaching. Under 358 kHz or 45 kHz US, faster calcite dissolution and diffusion occurred, leading to higher extraction yield (65.5 %). Citric acid enhanced dissolution by complexing Cu^2+^, but also Ca^2+^ and Mg^2+^ in fractions rich in clay minerals such as loam. Humic acid sodium salt at optimized alkali conditions may also mobilize Cu in alkali conditions. Sonication improved the extraction yield from the unseparated soil from 23.6 % in silent conditions to 30.5 % at 45 kHz and to 37.6 % at 358 kHz. Its effect was particularly pronounced on fine sand, with a yield increase from 18 % to 43 %. Sonication would trigger deagglomeration of bigger particles, and promote the transport of Cu-containing particles by micelles formed by humic species. This study highlighted the potential of high-frequency US in enhancing Cu extraction from a real soil, to a higher extent compared to lower frequency 45 kHz US.

## Highlights

• Cu depollution from a sandy loam soil is studied with citric and humic acids.
• 358 kHz US enhances Cu extraction more than 45 kHz US.
• Extraction yield after 1 h reaches 71 % with 0.2 M citric acid under 358 kHz US.
• US irradiation enhances dissolution/diffusion/transport phenomena.

## 1 Introduction

The development of anthropogenic and industrial activity makes polluted soil remediation an increasingly problematic environmental issue, as described in the 2030 Agenda for Sustainable Development at the United Nations Summit [1]. Over 10 million sites are polluted worldwide [2] with pollutants as diverse as polycyclic aromatic hydrocarbons, halogenated solvents, volatile organic compounds or heavy metals [3], [4]. In Europe, about 35 % of the contaminants found in soils are heavy metals [5]. Heavy metals are naturally present in the soils at trace levels due to rock weathering, biogeochemical cycles, volcanic eruptions and atmospheric deposition. However, industrial and agricultural activities, and consequential mining residues, fertilizers, pesticides, transportation emissions, increase their concentration above threshold values that cause problems for humans and ecosystems [6]. Once heavy metals penetrate the soil, they can bind to soil constituents through various processes (ion exchange, complexation, precipitation, etc.) [7]. Then, heavy metals may be mobilized in response to rocks weathering and environmental changes, that induce reactions with organic compounds and pH or redox changes. Such phenomena may provoke migration into groundwater [8], [9].

Copper is the third most widely used metal in the world [6]. The accumulation of copper in soil can result from various sources, such as mines, former wood processing sites, industries, and the agricultural application of copper-based fungicides [10]. The intensive use of fungicide treatments, particularly Bordeaux mixture (CuSO_4_ + CaO), increases the concentration of copper in the soil. It is noteworthy that 53 % of concentrations higher than 100 mg/kg are found in winegrowing areas [11].

The soil is a heterogeneous system that is composed mainly of a mineral fraction, accompanied by an organic fraction that generally amounts to less than 10 %. Metals are not distributed equally in the different fractions [12]. Besides, their binding strengths and consequently their bioavailability also highly differ between the different fractions. For this reason, it is of primary importance on the one hand to characterize the studied soils and on the other hand to determine the repartition of the contaminant in the different soil fractions and to measure the efficiency of decontamination not only in the original soil but also in its components. Two main approaches, chemical and physical, are commonly employed to characterize soil components and their interactions with pollutants. Chemical approaches focus on the element mobility and plant availability and rely on sequential extraction schemes [8]. The most widely used ones are that of Tessier et al. [9] and that developed by the European Commission, Standards, Measurements and Testing Programme (BCR) [13]. Several chemical treatments are applied successively, starting from a mild one, quantifying contaminants weakly bound to the solid phase and continuing with harsher treatments to target contaminants fixed more strongly. This is why sequential chemical extraction can be seen as an operational speciation method [14], [15]: the species is defined by the chemical conditions in which it is extracted. Its results are often difficult to interpret due to the diversity and complexity of the constituents, and not quantitative due to the non-selectivity of reagents for target phases, the re-distribution of analytes among phases during extraction, and incomplete extraction and precipitation of new mineral phases during extraction [15]. In contrast, the soil texture classification [16], [17] appears more straightforward, which is based on separating soil fractions according to particle size. No unique classification exists [17]. The AFNOR norm was used here [18] that defines sand as the particles > 50 µm (coarse sand > 2 mm, fine sand between 50 µm and 2 mm), silt as particles between 2 and 50 µm (coarse silt between 20 µm and 50 µm, fine silt between 2 µm and 20 µm) and clay as the granulometric fraction < 2 µm.

Many techniques have been used to treat polluted soils. The choice of the most appropriate one is based on cost, treatment duration, effectiveness, and generation of secondary by-products, potentially harmful for the environment. These techniques can be applied on-site, such as in situ direct decontamination of the soil or mobile units, or off-site in dedicated treatment facilities [19]. Two approaches are to be distinguished: technologies that retain pollutants in the soil and stop them from migrating, such as stabilization and solidification, and technologies that extract pollutants from the soil [20]. If thermal treatment is a highly effective and rapid method, it is also expensive and the application of high temperatures disrupts the ecosystem and damages the soil [21], [22]. Phytoremediation is an eco-friendly remediation method [23], [24], but its low extraction kinetics and the fact that it is limited to surface contamination up to a depth of one meter restrict its application at high scale. Finally, chemical remediation offers high extraction yields, fast treatment kinetics, and generally lower costs, and is therefore often considered a relevant choice.

Washing with a dedicated leachant is based on improving metals mobility [19], [25], [26]. Most studies on cationic heavy metal ions used solutions of organic or inorganic acids or of complexing agents [27]. Strong inorganic acids and in particular hydrochloric acid have been commonly utilized due to their high efficiencies [28], [29], [30]. At low pH, protons interact with soil surface sites, facilitating the extraction of metal cations through ion exchange and the dissolution of e.g. metal oxides [31]. However, the application of strong acids increases soil acidity and subsequently disrupts the microorganisms present in the soil, requiring subsequent treatment [32], [33]. In order to avoid such an effect, gentler and more environmentally friendly cleaning agents have been considered, in particular low molecular weight organic acids like citric acid [34] that combine the action of protons to complexing properties, other complexing agents like EDTA [35] and humic substances [36], [37], [38]. Organic acids and humic substances present the advantage of being natural and easily biodegradable. For instance, He et al. [34] showed that up to 45 % of Cu was removed from an artificially contaminated soil at a citric acid concentration of 0.2 M, and Ke et al. [35] significantly enhanced the extraction process from an industrial soil by combining 0.5 M citric acid with 0.05 M EDTA (pH 3), achieving over 80 % recovery of Cu, Ni and Zn in 6 h with a 1:10 soil to leachant mass ratio [39]. As for humic substances, they allowed to remove 82.8 % Cu from an artificially contaminated soil collected in an agricultural area, with a humic substances concentration of 2335.4 mg/L, a treatment duration of 12 h and a solid-to-liquid ratio of 1:30 [40].

Several studies showed that combining ultrasound (US) with leaching could significantly enhance pollutant removal from soil. Indeed, the coupling of ultrasonic irradiation, washing and mechanical stirring could lead to higher extraction yields, shorter treatment durations and smaller secondary wastewater generation [29], [34], [41], [42], [43], [44], [45], [46], [47], [48], [49], [50]. This improvement was attributed to acoustic cavitation, the formation and violent collapse of cavitation bubbles, that accelerates microscale mixing via shock waves and microjets generated by bubble collapse [42], [51], [52]. For example, Kim et al. [53] reported that copper extraction yield in 0.3 M HCl increased from 31.4 % with mechanical stirring alone to 53.6 % when 28 kHz ultrasound was applied. Similarly, Park et al. [43] and Son et al. [42] observed that 0.5 M HCl washing alone led to copper extraction yields of 47.2 % [43] and 51 % [42], and that these extraction yields increased up to 76.2 % [43] and 62 % [42] in the presence of an ultrasonic irradiation. Yet, several studies [29], [41], [54], [55] also underlined that a beneficial impact of ultrasound was not observed for all pollutants nor for all soils and washing solutions, and that sonication may even lead to lower desorption. In particular, under low frequency (20–50 kHz) ultrasound irradiation, the fragmentation of soil particles resulted in the creation of new surfaces where metals could re-adsorb [29], [44], [54]. This negative effect was reduced at ultrasonic frequencies higher than 200 kHz [29].

The aim of the present study is to evaluate the contribution of low and high frequency US in soil remediation by evaluating total Cu removal and its extraction kinetics. A natural soil artificially contaminated by copper is considered. Copper release under leaching is monitored both on the entire soil and on two of its granulometric fractions (fine loam and fine sand) to evaluate the decontamination efficiency and allow better comparison with other soils. Three different washing solutions are considered: a strong inorganic acid (HCl) as a reference, and two organic biosourced reagents, citric acid and a sodium salt solution of humic acid. Soil washing experiments are conducted under non-sonicated conditions (using only mechanical stirring), as well as at two ultrasonic frequencies: 45 kHz (low frequency) and 358 kHz (high frequency).

## 2 Materials and methods

### 2.1 Soil sample and chemicals

The studied soil was collected from the surface (20–––30 cm) of a garden located in Saint-Laurent-des-Arbres, Gard, France. After collecting the soil, the leaves and stones were removed and the soil was sieved at 2 mm.

All experiments were performed using milli-Q-grade ultrapure water (18 MΩ.cm at 25°C), air (O_2_ 20 vol%, N_2_ 80 vol%, Air Liquide) and analytical-grade reagents: copper sulfate (CuSO_4_, purity 99 %), hydrochloric acid (HCl, 37 %), citric acid (C_6_H_8_O_7_, purity 99 %), humic acid sodium salt (technical grade), nitric acid (HNO_3_, 65 %) and hydrofluoric acid (HF, 40 %) purchased from Sigma-Aldrich, methylene blue (C_16_H_18_CIN_3_S, 1 %) purchased from Thermo Scientific.

### 2.2 Analytical methods

Particle size distribution was measured using laser granulometry (CILAS 1090 particle size analyzer). ICP-OES (Thermo Scientific iCAP 6000 spectrometer) was used to determine the concentration of Cu in liquid samples. Scanning Electron Microscopy (SEM, VEGA3, TESCAN) was used to determine the morphology of the soil particles. The chemical composition of the soil was measured by energy dispersive X-ray spectroscopy (EDX, Bruker XFlash® 5010 SDD). Further details regarding the nature of the minerals constituting the soil were provided by X-ray diffraction pattern (2θ range between 2 and 70°, step size 0.013° and step time 2 s) using the device PANalytical X’ Pert Pro – XRPD. This instrument was equipped with a Cu source in Bragg-Brentano geometry. The pH of the soil was determined in a mixture of soil: Milli-Q-water at a ratio 1:10 with a METTLER TOLEDO pH meter following NF EN ISO 10390.

Nitrogen adsorption–desorption isotherms were measured at − 196°C using a AntonPaar Nova 800 surface area and pore size analyzer. The samples were degassed at 90°C for 24 h before analysis. The Brunauer-Emmett-Teller (BET) method was used to calculate the specific surface areas.

Thermogravimetric analysis (TGA) was conducted on a Mettler Toledo TGA/ DTA device coupled with a mass spectrometer to monitor water and carbon dioxide in the gas phase. The soil sample (28.9 mg of unseparated soil, 69 mg of coarse sand, 29.6 mg of fine sand, 19.5 mg of coarse silt, 27.8 mg of fine silt or 10.7 mg of clay) was placed in a platinum pan. Samples were first heated to 200°C, held at this temperature for 10 min, then heated up to 700°C at a rate of 10°C/min, held at 700°C for 10 min, and the temperature was finally further increased to 900°C at the same heating rate (10°C/min). The whole process was performed under an air flow at 20 mL/min.

The amount of dissolved humic acid sodium salt was estimated by Total Organic Content (TOC) measurement (Shimadzu TOC-5000A TOC-meter) after filtration. The experimental uncertainty is estimated to 10 %.

### 2.3 Separation of the soil constituents

Soil fractions were separated according to particle size using the AFNOR NF X 31–107 norm [18] that defines sand as the particles > 50 µm (coarse sand > 2 mm, fine sand between 50 µm and 2 mm), silt as particles between 2 and 50 µm (coarse silt between 20 µm and 50 µm, fine silt between 2 µm and 20 µm) and clay as the granulometric fraction < 2 µm. This protocol consists in separating particles according to their size: 13 g of soil were sieved to 2 mm and then suspended into 150 mL demineralized water and stirred for 12 h. Subsequently, the solution was sieved using 3 different sieves: 2 mm, 50 µm and 20 µm. The fraction < 20 µm (mixture of fine silt fraction and clay fraction) was introduced into a test tube and completed to 1 L with demineralized water. After sedimentation, the fine fraction < 2 µm (clay) was extracted by suction from the first 10 cm of the surface using a pump. Following their final drying at 80°C for 24 h, the different fractions were weighed using an analytical balance. The type of soil under study was then identified from the mass ratios of these fractions using the U.S. Department of Agriculture (USDA) texture triangle [16].

### 2.4 Adsorption capacity and cation exchange capacity

The methylene blue assay (standard norm NF P94-068) was used to determine the specific surface area of the soil particles and infer from it the nature of the clay in the soil (expanding clays have a higher adsorption capacity). A 10 g.L^−1^ methylene blue solution was added in increments of 10 mL, 5 mL and then 2 mL to 50 g of dry soil mixed with 500 mL of demineralized water while stirring at 400 rpm. After each addition, the suspension was stirred for 1 min, and then a drop of suspension was taken and placed on a filter paper until a light blue peripheral halo appeared, that indicated all sorption sites of the soil were saturated. The MB value is defined as the mass (in g) of methylene blue sorbed on 100 g of dry soil, i.e. the mass of methylene blue that had been added up to the appearance of the blue halo.

The cation exchange capacity (CEC, in Eq/100 g) can be calculated using Equation (1) [56] where 374 g/mol is the molar mass of methylene blue, under the assumption that methylene blue is entirely adsorbed in its monovalent form.

(1) CEC=MB/374

### 2.5 Contamination of the soil

**Fig. 1:**
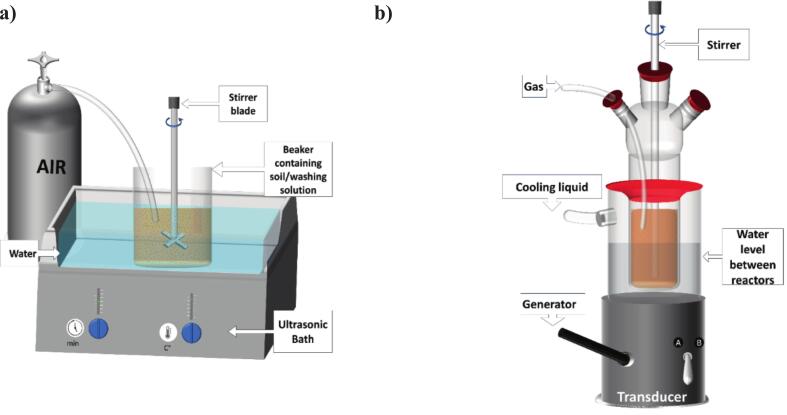
A) ultrasonic bath setup for decontamination at 45 kHz, b) Sonolysis setup at 358 kHz with no direct contact between the transducer and the soil/washing solution mixture.

For contamination, 100 g of unseparated soil were first stirred in 1 L of ultrapure water for 7 days at 235 rpm and a constant temperature of 25°C to ensure equilibration of the soil with the solution (see Fig. 1-SI). After 7 days, 1 mL of copper sulfate solution (10 g.L^−1^) was added and the mixture was stirred again for 7 days under the same conditions. Ultimately, Büchner filtration was used to separate the liquid from the solid, and the solid was dried at 80°C to avoid modifying the structure of the clays.

Microwave mineralization (Anton Paar, Multiwave 5000) of the soil and of its granulometric fractions was performed to quantify the amount of fixed Cu. In a microwave vessel, 0.1 mg of sample was digested by 10 mL acid mixture (6 mL of HNO_3_ 65 %, 2 mL of HCl 37 % and 2 mL of HF 40 %). The vessels were closed and the temperature was increased to 200°C over 20 min and maintained at that level for 10 min. The vessels were then let to cool at room temperature and diluted to a final volume of 50 mL with MQ water. Finally, the copper concentration was measured by ICP-OES to calculate copper adsorption capacity Q_0_ (mg(Cu)/g_solid_). The total copper contamination in the soil was 100 ppm.

### 2.6 Decontamination

Decontamination experiments were carried out in two different setups using three different washing solutions: hydrochloric acid HCl (0.1 M), citric acid (0.2 M) and humic acid sodium salt (4.5 g.L^−1^).

In order to distinguish the contributions in citric acid efficiency of its complexing properties and of the acidic pH, decontamination experiments were carried out with HCl solutions of pH 2.5. The pH was measured continuously and kept constant during the whole experiment by adding HCl 0.5 M when needed.

**Fig. 2:**
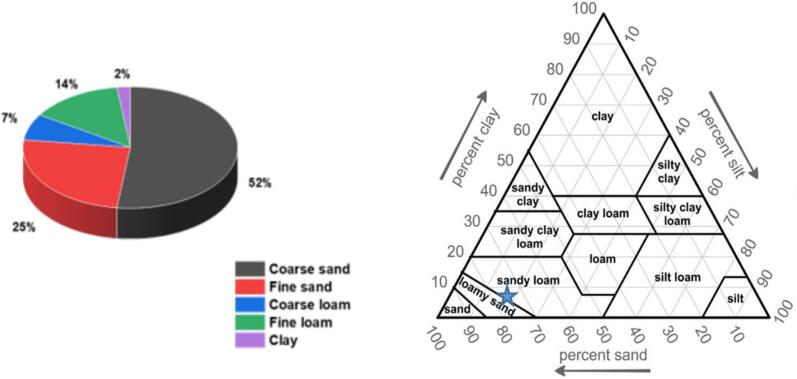
A) distribution of the different granulometric fractions constituting the soil b) type of the soil referring to the usda triangle [16].

The optimum concentration of humic acid sodium salt (NaH) and the optimum pH were determined by stirring 1 g of soil with 10 mL of solution at concentrations of NaH ranging from 0 to 6 g.L^−1^ for 24 h (Fig. 2-a-SI in Supporting Information).

Experiments with low frequency (45 kHz) ultrasound (Fig. 1-a) were performed in a beaker containing the soil sample and the washing solution stirred by mechanical agitation at 450 rpm, placed in an ultrasonic bath (USC 100 T, 45 kHz, 30 W electrical power). The absorbed acoustic power was determined by calorimetry (5.3 W). An air bubbling of 80 mL.min^−1^ was applied and the temperature was maintained around 27°C during the experiment by adding ice to the water containing the beaker.

High-frequency sonolysis was performed at 358 kHz in a “no direct contact” set-up (Fig. 1-b), under mechanical stirring at 450 rpm. In this case, experiments were carried out in a small glass reactor immersed in water contained in a second thermostatically controlled reactor placed on a transducer (L3C ELAC NAUTIK, 25 cm^2^, providing two frequencies: A = 345 kHz and B = 1045 kHz) connected to a 125-W generator (T&C Power Conversion, Inc.). The absorbed acoustic power was determined by calorimetry (29 W). The sample temperature was kept around 28°C ± 0,5°C. For comparison, “silent mode experiments” were performed under the same conditions but without the application of ultrasound.

In all experiments, the mass of soil was 5 g and the volume of solution was 100 mL. After contacting the soil with each washing solution, samples (approximately 2 mL) were taken every 2 min, filtered (with 0.45 µm filters) and the concentration of copper was measured using ICP-OES. The extraction yield (%) was calculated by Equation (2):

(2) $$ExtractionYield\left(\%\right)=\frac{{\left[Cu\right]}_{t}\times V}{Q_{0}\times m_{soil}}\times 100$$

With [Cu]_t_ the copper concentration in solution at time t (mg/L), *V* the volume of the washing solution (in L), m_soil_ the mass of soil (in g) and *Q*_0_ the initial copper loading per unit mass of soil (in mg/g).

### 2.7 Desorption kinetics models

Desorption models are commonly implemented to assess mechanisms involved in depollution. Kinetics can be modelled as pseudo first order, pseudo second order, parabolic or Elovich equations. In this study, the parabolic model of Equation 3 was used to fit the decontamination curves:

***[Cu]_t_ = [Cu]_0_ + k_diff_.t^0^****^.5^* ***(3)***.

With k_diff_ the diffusion constant, [Cu]_0_ the initial Cu concentration in solution, in mg/kg, and [Cu]_t_ the Cu concentration in solution at time t. Besides, an initial Cu release rate, V_0_, was estimated via the first measured extraction yield divided by the time of the first measurement, which was 2 min in our case.

The relevance of the model was checked using two criteria: the usual R^2^ coefficient and the standard error SE defined by Equation (4).

(4) $$SE={\left[\frac{{\sum \left({\left[Cu\right]}_{t}-{\left[Cu\right]}_{mod}\right)}^{2}}{\left(N-2\right)}\right]}^{0,5}$$

The standard error was calculated from the measured ([Cu]_t_) and calculated (${\left[Cu\right]}_{mod}$) concentrations of Cu in the solution at time t, with N representing the number of measurements performed.

### 2.8 Simulation using PhreeqC

Since metals can exist in different forms (dissolved ions or organic complexes, exchangeable ions, precipitates or coprecipitates), it is essential to consider their speciation [19]. PhreeqC Interactive 3.7.3 software was used with Minteq V4 database. Only the liquid phase was considered and the copper concentration was fixed at 5 mg/L or 7.9.10^-5^ M, corresponding to complete decontamination of 5 g of contaminated soil in 100 mL of solution. The concentration of HCl (0.1 M) and citric acid (0.2 M) were set to the values used in the experiments, and pH variations were applied while monitoring the speciation of the species formed in solution.

## 3 Results and discussion

### 3.1 Characterization of the soil before contamination

**Fig. 3:**
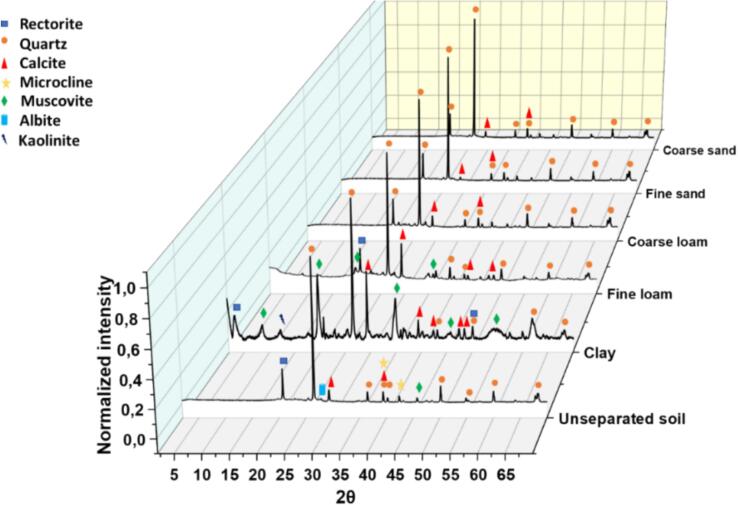
X-ray diffractograms of the soil fractions (before contamination with copper).

**Fig. 4:**
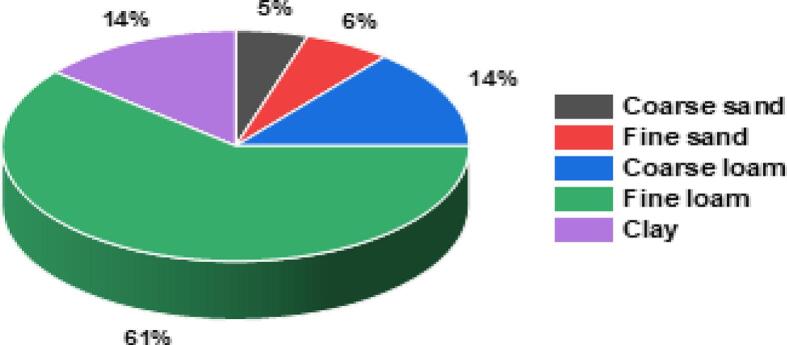
Distribution of copper in the different soil fractions.

The soil is a heterogeneous system. The particle size distribution is large, as indicated by granulometric analysis (Fig. 3-SI) and SEM measurements (Fig. 4-SI). Following application of the AFNOR analytical protocol, the proportions of the various soil fractions were determined: 52.0 ± 5.0 % coarse sand, 24.5 ± 2.5 % fine sand, 7.0 ± 0.8 % coarse silt, 14.4 ± 1.7 % fine silt and 2.2 ± 0.8 % clay (Fig. 2-a). According to the mass percentages of sand (coarse + fine: 77 ± 7 %), loam (coarse + fine: 21 ± 2 %) and clay (2 ± 1 %), the USDA triangle showed that the studied soil was sandy-loam type (blue star in Fig. 2-b).

Soil characteristics are presented in Table 1. The conductivity of the soil–water solution was monitored over 14 days (Fig. 1-SI). It increased strongly in the first 2 days and was approximately constant after 3 days, stabilizing at 68 ± 7 µS/cm. The soil MB adsorption capacity was calculated to be 0.90 ± 0.05 g of methylene blue per 100 g of soil. The cation exchange capacity (CEC) was calculated with Equation (1) as 2.41 ± 0.12 mEq per 100 g of soil. The amount of copper initially present in the soil was estimated by mineralization and was found to be below the detection limit of ICP-OES (2.10^-4^ mg (Cu)/g).

**Table 1:** Characterization of the studied sandy loam soil: ph, conductivity, adsorption capacity, cation exchange capacity and initial amount of cu in soil.

**Type of the soil**	**pH**	**Conductivity after 24 h**	**MB adsorption capacity**	**Cation Exchange Capacity (CEC)**	**Initial amount of Cu**
		µS.cm^−1^	g of methylene blue /100 g of soil	mEq/100 g of soil	mg(Cu)/g
Sandy loam	8	68 ± 7	0.9 ± 0.05	± 0.12	2.10^-4^

Specific surface areas were determined by BET analysis for fine sand (1.2 ± 0.1 m^2^/g), fine loam (15.8 ± 0.8 m^2^/g) and clay (36.8 ± 1.8 m^2^/g). As is usually the case, the specific surface area increases when the particle size decreases.

Capillary XRD characterization was applied to the complete soil and its five fractions (coarse sand, fine sand, coarse loam, fine loam and clay). As shown in Fig. 3, a predominance of quartz and calcite is identified in all soil fractions. The high intensity of the corresponding peaks makes it difficult to detect the peaks of other phases and only quartz and calcite are seen in sand and in coarse loam. In fine loam, muscovite and rectorite peaks are also noticed. Clay seems to consist in many more phases. Thus, the different soil fractions do not only differ by size but also by composition.

Thermogravimetric measurements were performed to quantify the organic content in each soil fraction. Table 1-SI in Supporting Information presents for each fraction on the left-hand side the time-evolution of sample temperature, sample mass loss and, to highlight abrupt mass changes, differential mass loss, and on the right-hand side the time-evolution of MS intensities corresponding to water and carbon dioxide. Mass losses are observed in four different temperature intervals that correspond according to [57] to losses of water (T < 200°C), organic carbon (200 < T < 430°C), clay minerals (430 < T < 590°C) and carbonates (T > 600°C), and are summarized in Table 2.

**Table 2:** Mass losses of the different soil fractions (% of the initial mass) in different temperature intervals, determined by TGA.

Soil fraction	Δm < 200°C Water	Δm 200-430°C Organic carbon	Δm 430-590°C Clay minerals	Δm > 600°C Carbonates
Total soil	0.92	1.66	0.82	3.57
Coarse sand	0.04	0.33	0.18	2.19
Fine sand	0.21	1.56	0.78	3.20
Coarse loam	0.80	2.88	1.40	4.23
Fine loam	3.24	5.15	2.96	5.50
Clay	4.78	6.31	4.44	0.65

Coarse sand hardly contains water and organic matter. In smaller soil fractions, the four successive mass losses are clearly visible. The water content amounts from 0.2 % in fine sand to 4.8 % in clay. Estimated Δm corresponding to clay minerals are in agreement with XRD spectra and the increasing presence of clay minerals in smaller soil fractions. Similarly to clay minerals, organic matter is preferentially present in loam and clay and its amount increases with decreasing fraction size. These higher amounts of organic carbon in finer fractions may be attributed to higher surface-to-volume ratios, taking into account the very low porosities determined for the different fractions. Besides, in sand and in coarse loam only quartz and calcite were identified in XRD spectra, phases that hardly bind carbon [58]. On the other hand, the increasing presence of clay minerals in the smaller fractions is favourable for organic carbon fixation [59].

### 3.2 Characterization of the soil after contamination

Once characterized, each soil fraction was subjected to mineralization to identify the distribution of the copper contamination within the matrix. Fig. 4 exemplifies this distribution and Table 2-SI in Supporting Information summarizes the mass of each fraction in g and its measured copper content in mg/g and in mg. As can be seen from the copper loads, that amount from 0.01 to 0.5 mg/g, copper is not distributed homogeneously in the different soil fractions. Although the sand fraction represents 77 % of the total soil mass, it contains only 11 % of the total copper. In contrast, the main copper carriers are the loam and clay fractions, even though they are less abundant overall. Notably, fine loam, representing only 14 % of the soil, retains most of the contamination, accounting for 61 % of the total copper content. These findings highlight the preferential accumulation of copper in finer soil particles. In the literature [60], [61] it was demonstrated that heavy metals preferentially accumulate in fine size fractions: the highest concentrations of heavy metals were generally observed in the clay fraction, followed by loam and then sand. The strong tendency of clays to retain metals was attributed to their larger specific surface area, negatively charged surfaces and higher organic matter content [62], [63]. Also, Bradl [64] concluded that organic matter was a sink for Cu in soils at pH > 5, together with carbonates. In general, the trend observed here correlates with the organic matter and carbonate contents that increase when the soil fraction size decreases (except for carbonates whose content is very low in clay), which explains the increasing affinity of Cu when the soil fraction size decreases.

### 3.3 Decontamination of the unseparated soil with HCl

**Fig. 5:**
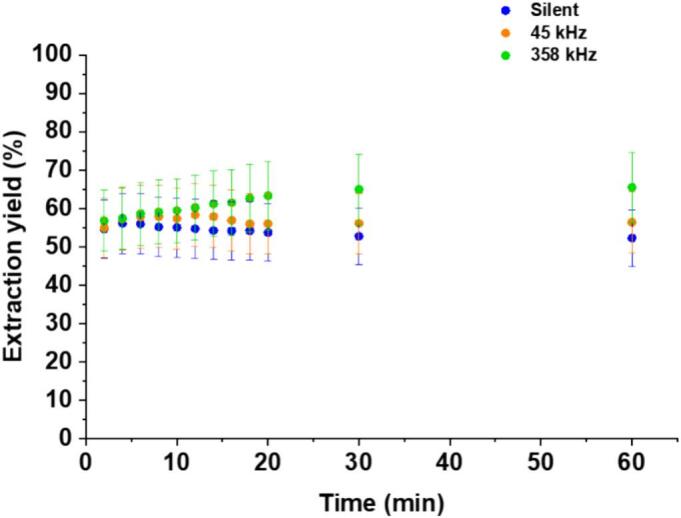
Evolution of Cu extraction yield from the unseparated soil by washing with 0.1 M HCl under silent conditions, 45 kHz and 358 kHz US irradiation, m/V ratio = 5 g/100 mL and final solution pH = 2.

To minimize the negative effects of hydrochloric acid on the soil, a low concentration of 0.1 M was chosen. Washing the soil with HCl resulted in a pH around 2. At this pH, Cu is only present as Cu^2+^, as indicated in Fig. 5-SI that shows the Cu (II) speciation diagram calculated using PhreeqC software for a solution containing 1.57 × 10^–4^ M of Cu (II). Fig. 5 presents the evolution of the decontamination yields in 0.1 M HCl under silent, low frequency and high frequency US conditions. The decontamination process shows a fast initial phase: almost half of the pollutants has been removed in the first 2 min, with no difference between the three conditions, indicating that dissolution by HCl is responsible for the initial decontamination. At this pH, at least carbonates are dissolved, releasing the corresponding Cu part in solution. No subsequent evolution is seen under silent conditions. Under US irradiation at 358 kHz, the extraction efficiency then slowly increases with time until it reaches a plateau around 65 % after 30 min. This higher yield under US is usually attributed to the mechanical effects of acoustic cavitation. These effects were recently shown [29], [48], [65] to trigger the transfer of metal pollutants from the fractions of Tessier protocol that are more difficult to mobilize (oxide and residual fractions) to more accessible fractions (namely exchangeable, acid-extractable and reducible fractions), thus allowing an increase in the desorption yield. Interestingly, a more complex behaviour is observed at low-frequency US (45 kHz) compared to high-frequency US: while in the first 12 min the yield follows the curve obtained at 358 kHz, indicating enhancement by cavitation, it then starts to decrease, almost to the level observed under silent conditions. Such a negative contribution of low US frequency in the decontamination of soils was recently reported [29] on vermiculite and explained by the strong fragmentation of the particles, resulting in the creation of new sorption sites that can trap back part of the released ions. In the first 12 min the fragmentation is still limited and only the positive impact of enhanced desorption is seen (attributed to transfer of Cu to more accessible fractions); then fragmentation becomes more and more pronounced, creating more and more new sorption sites where some freshly desorbed Cu can be trapped back.

### 3.4 Decontamination using a citric acid solution

**Fig. 6:**
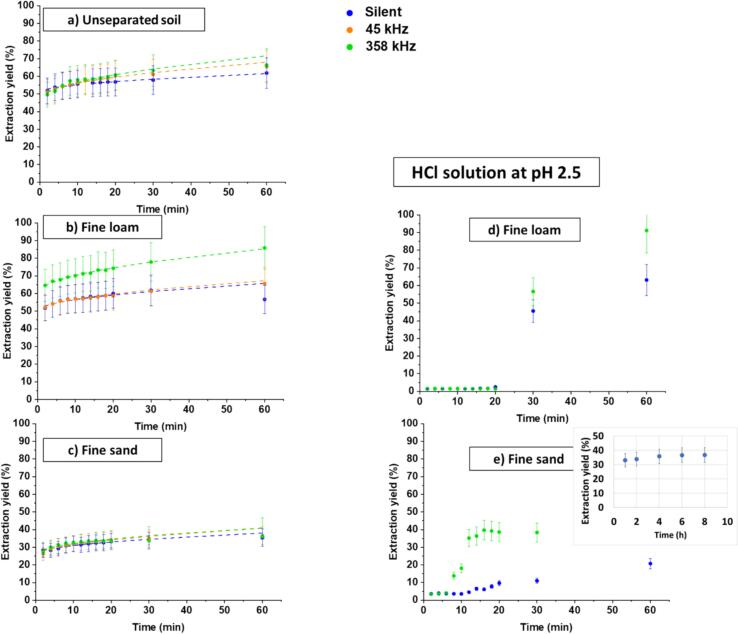
Kinetics of Cu extraction during washing with 0.2 M citric acid for a) unseparated soil, b) fine loam, and c) fine sand under silent conditions, in a 45 kHz ultrasonic bath and under high-frequency ultrasound at 358 kHz. Experiments were conducted at a solid-to-liquid ratio of 5 g/100 mL and a final solution pH of 2.5. d) and e) show Cu extraction kinetics during washing with HCl solution at pH 2.5 for fine loam and fine sand, respectively, under silent and 358 kHz ultrasonic conditions, using the same solid-to-liquid ratio. Dashed lines correspond to parabolic equation fits.

Decontamination with citric acid was performed on the unseparated soil and on two soil fractions: fine loam and fine sand. The fine loam fraction was selected as it stores the majority of the pollution (61 %). On the other hand, the soil being made up of 77 % sand, the fine sand fraction was also considered. A citric acid concentration of 0.2 M was chosen, which corresponds to a measured pH in the presence of soil of 2.5. According to a simulation performed using PhreeqC software for these experimental conditions and a total Cu concentration in solution taken as the one that would correspond to total extraction (Fig. 6-SI), five species are present in solution: CuH_2_(citrate)^+^ (50 %), Cu^2+^ (27 %), CuH(citrate) (17 %), Cu(citrate)^-^ (4 %) and Cu(citrate)_2_^4-^ (2 %).

Fig. 6a shows the decontamination with citric acid of unseparated soil. The general trend is comparable to that observed with HCl: a very fast initial increase reaching 51.7 % after 2 min followed by a slow increase and a plateau, with final extraction yields between 61 % in silent conditions and 66 % under high frequency US. The effects of sonication and of the US frequency are the same as with HCl. Similar curve shapes are obtained for fine loam (Fig. 6-b) and fine sand (Fig. 6-c), with 1-h yields in silent conditions of 62 % with fine loam and 36 % with fine sand. Sonication with high-frequency ultrasound resulted in a better extraction, especially in the case of fine loam, with a 39 % (resp. 30 %) increase in extraction yield after one hour compared to silent (resp. low-frequency US) conditions.

The values of initial release rates V_0_ are summarized in Table 3-SI, that also presents 1-hour extraction yields. Except in the case of fine loam where high-frequency US leads to a 30 % increase in V_0_, no significant impact of US is observed on the initial decontamination rate: it is around 25.4 %.min^−1^ in the case of unseparated soil whatever the experimental conditions and around 13.7 %.min^−1^ with fine sand. The fast initial decontamination indicates release of easily accessible Cu, which at pH 2.5 may be Cu bound to carbonates. This interpretation is supported by the factor 2 difference between carbonates contents in fine loam and in fine sand, factor 2 that is also observed in V_0_. A similar impact of the carbonate content on the metal release was reported by Jalali et al.[66]. The very fast acidic dissolution of carbonates explains the absence of impact of sonication except in fine loam at 358 kHz.

Following the fast initial Cu release, the extraction slows down, indicating that another mechanism becomes predominant: Cu is no longer solely released by the very fast dissolution of calcite but also from other sites, which implies desorption, internal diffusion in the soil, diffusion in the bulk and complexation. This intricate mechanism was modelled using parabolic equation (Eq. 3). This model appropriately fits experimental curves, as seen in Fig. 6 (dashed lines) and indicated by the high R^2^ values and low SE values in Table 3**-SI.** The key parameter in Eq. 3 is the diffusion coefficient k_diff_. A clear increase in k_diff_ is observed in the presence of US, especially at high frequency. In the unseparated soil, k_diff_ doubles at 45 kHz and almost triples at 358 kHz, in agreement with the well-known accelerated diffusion under US, possibly counteracted by readsorption on sites created by fragmentation or disaggregation at low frequency. A similar trend, though less marked, is observed for fine loam. On fine sand, a small increase is noted only at high frequency. Interestingly, the evolution of k_diff_ values in the unseparated soil case, and in particular the strong influence of US irradiation, cannot be explained neither by that of its main fraction, sand, nor by that of loam, the fraction bearing most of the contamination. It can thus be suggested that it may be linked to the minority clay fraction. This fraction is poor in carbonates but rich in organic carbon and contains 14 % of the fixed Cu. A recent study reported ultrasonic improvement of Zn/Ni desorption from vermiculite, especially at high frequency US [29].

Fig. 6-d and 6-e present the time-evolution of Cu extraction yield when washing with HCl solutions of pH 2.5 for fine sand and fine loam under silent conditions and high-frequency US irradiation. While extraction yields after 1 h are very similar to those obtained with citric acid at the same pH value (except in the case of fine sand under silent conditions), the kinetics are very different. Notably, contrary to the citric acid cases, the extraction yields are very low in the first minutes, and only start to increase after some induction period, of about 6–12 min for fine sand and 20–30 min for fine loam.

This delay can be attributed to the kinetics of dissolution of minerals (in particular carbonates) [67]. While dissolution is very fast at pH 2 (HCl 0.1 M), it is slower at pH 2.5. This difference may be attributed to a 3.2 times lower concentration of protons in solution [68]. A smaller delay is observed under high-frequency US irradiation (6–8 min for fine sand, vs. 14 min under silent conditions) due to acceleration of the dissolution kinetics by the mechanical effects of cavitation. To investigate whether the impact of US is only on the kinetics or also on the final yield, the experiment at pH 2.5 under silent conditions was prolonged to 8 h on fine sand (insert in Fig. 6-e). A plateau is reached after 4 h, at a yield of 35.7 %. Under US, the plateau is reached at the same final value after 30 min. Thus, sonication increases the kinetics but does not affect the final yield. Similarly, the presence of citric acid does not increase the final yield but increases the kinetics by complexing various cations: Cu^2+^ but also Ca^2+^ and Mg^2+^, thus increasing dissolution rates. This dissolution mechanism is efficient for the decontamination of fractions rich in clay minerals, such as fine loam. On the other hand, it is less efficient for sand that hardly contains them. In sand, Cu is more probably bound to silica, that is less sensitive to citric acid dissolution.

### 3.5 Decontamination using a solution of humic acid sodium salt

When mixing humic acid sodium salt (NaH) with water, the pH naturally evolved to a constant value of 10. At this pH value, Cu (II) is mainly in the form of Cu(OH)_2_ (see speciation diagram calculated with PhreeqC in Fig. 5-SI), which has a very low solubility (around 10^-5^ g/L). The results show that despite this very low solubility, copper extraction takes place in the presence of humic acid sodium salt, and the extraction yield increases with NaH concentration until it reaches a plateau for [NaH] between 4 and 5 g.L^−1^ (Fig. 2**-**a-SI). Copper extraction cannot be attributed to the dissolved part of humic acid, which is very low: Total Organic Carbon measurements indicate a dissolved carbon below 4 mg/L whatever the pH between 4 and 12 (see Table 4-SI in Supporting Information). Instead, copper extraction is linked to the colloidal properties of humic substances. Indeed, above the critical micelle concentration, the excess of humic species form pseudo-micelles [69], [70] where hydrophobic moieties form a core insulated from the aqueous phase by outward oriented hydrophilic moieties [71]. Such micelles formation could increase the mobility of metals or any other highly hydrophobic pollutant and their removal from soil [37].

After fixing the concentration at 4.5 g.L^−1^, experiments were conducted at different pH values under identical conditions (Fig. 2-b-SI in Supporting Information). The extraction was very low at pH = 2 and 4, and reached 39.5 % ± 14 % above pH 6. This tendency is consistent with the literature: Kulikowska et al. [69] and Damian et al. [72] showed maximal copper removal at alkaline pH, compared to acidic/neutral pH, contrary to other leachants, for which higher copper extraction was obtained at acidic pH. At low pH, the protonation of carboxylic functional groups induces a decrease of humic acid ability to complex Cu (II) ions, whereas at alkaline pH, complexation of Cu (II) may be linked to the absence of competition with H^+^ for fully deprotonated carboxylic sites. Phenolic hydroxyl groups tend to dissociate at pH between 8 and 13. As described by Wang et al.[73] increasing pH may also induce mobilization of metals by humic substances. Thereafter, a pH of 10 was chosen.

**Fig. 7:**
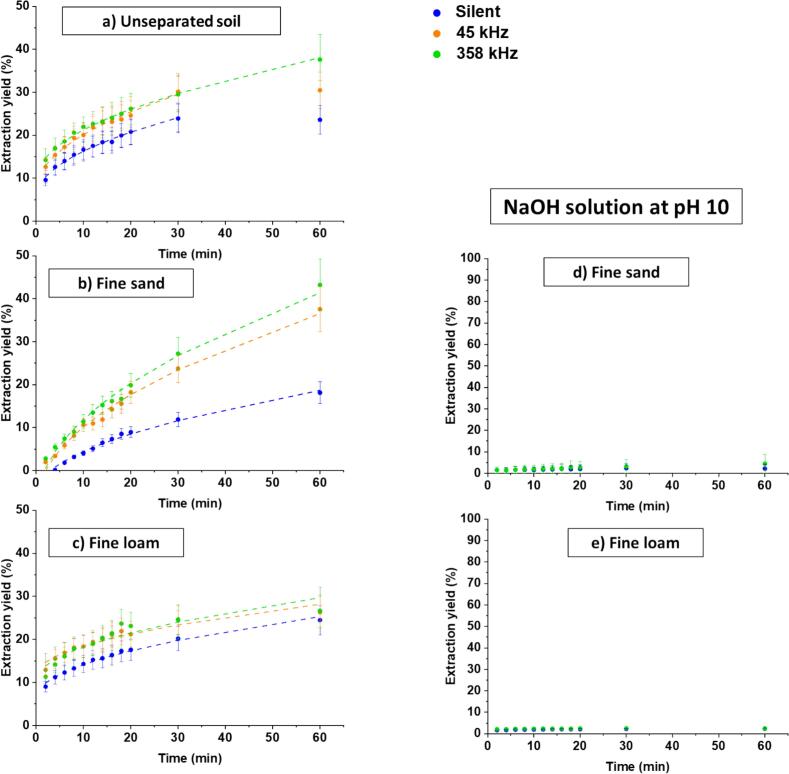
Kinetics of Cu^2+^extraction during washing with 4.5 g.L^−1^ of sodium salt humic acid for a) unseparated soil, b) fine sand, and c) fine loam under three different experimental conditions silent, 45 kHz and 358 kHz. Experiments were conducted at a solid-to-liquid ratio = 5 g/100 mL and a final solution pH of 10. d and e show kinetics of Cu^2+^extraction during washing with NaOH solution at pH 10 for fine sand and fine loam, respectively, under silent and 358 kHz conditions, using the same solid-to-liquid ratio. Dashed lines correspond to parabolic equation fits.

At the chosen pH of 10, copper only exists as Cu(OH)_2_ hydroxides, as calculated by PhreeqC software (Fig. 5-SI). It was checked that at this pH, hardly any Cu was removed from the soil in the absence of humic acid sodium salt (Fig. 7-d&e), indicating that, in the present case, organic matter hardly dissolved. In contrast, in its presence, extraction yields of the order of 30 % were reached after 1 h (Fig. 7-d and 7-e).

It is to be noted that the extraction yield continued to increase after 1 h. This, combined to the fact that operating conditions (in particular, soil-to-solution ratio) remain to be optimized, may explain why results obtained here are not as good as those reported by Lim et al. [74] who reached 69 % Cu removal. Other authors noted very slow decontamination kinetics and compensated for it by successive washings: 10 24-h extractions by humic acid solution allowed the removal of “only” 41 % of Cu from a loamy sand calcareous soil contaminated at 1 000 ppm Cu [36].

Sonication improves extraction yields, that range after 1 h treatment from 23.6 % in silent conditions to 37.6 % at 358 kHz US (Fig. 7-a). The curve seems to reach a plateau after 30 min in silent conditions and at 45 kHz, whereas the extraction yield keeps increasing at 358 kHz. This behavior may be explained by the fact that applying high frequency US would help to reduce Cu exchangeable fractions, but also to desorb Cu linked to dissolved organic matter, carbonates or other oxides, without the readsorption typically observed at low frequency [29]. The higher extraction yields obtained with HCl and citric acid compared to NaH may be attributed to calcite dissolution at acidic pH [75].

To clarify the impact of the soil composition on the extraction yield, further decontamination experiments were performed on the soil fractions fine sand and fine loam (Fig. 7-b and 7-c). Considering the fine loam fraction, the extraction yield reaches 24.4 % after 1 h in silent conditions, close to the unseparated soil value. Sonication allows only a slight increase in the extraction yield: 26.6 % for both frequencies. In the case of fine sand, the silent conditions decontamination is low compared to the unseparated soil and fine loam (19 % after 1 h). The difference in behavior of fine loam and fine sand fractions is linked with their mineral compositions and in particular their relative contents in carbonates and clay minerals (Table 2), with copper having a high affinity towards carbonates and a low towards clay minerals [64]. Besides, the higher dissolved organic matter content in fine loam than in fine sand may also hinder Cu desorption.

Decontamination of fine sand increases to 37.5 % at 45 kHz and 43.2 % at 362 kHz, i.e. values similar to those obtained on the unseparated soil. Thus, the effect of ultrasound is very pronounced in fine sand. The same trend is observed on initial release rates. A higher initial decontamination rate is observed on the unseparated soil at high-frequency ultrasound (V_0_ = 7.1 %.min^−1^) compared to low-frequency US (V_0_ = 6.8 %.min^−1^) and silent conditions (V_0_ = 4.8 %.min^−1^). The initial decontamination rates using humic acid are much higher than using citric acid (around 25.4 %.min^−1^), which agrees with the suggested mechanism of acidic dissolution of calcite with citric acid, that does not take place with NaH. In fine sand, V_0_ is extremely low in silent conditions and strongly increases in the presence of US (0.5–0.7 %.min^−1^). It may be suggested that the fine loam fraction is small enough to form micelles with humic acid, whereas sand would be too big. Sonication would trigger deagglomeration of bigger particles, which would consequently promote the transport of Cu-containing particles by micelles. This impact is visible on fine loam and huge on sand. Due to the low amount of Cu present in the sand compared to the other soil fractions, the impact on the unseparated soil is intermediate. No striking effect of US is observed on k_diff_.

## 4 Conclusion

Due to increased industrial and agricultural applications, Cu accumulation in soils calls for the development of efficient and environmentally friendly depollution techniques. This study focuses on a real sandy loam soil, in which Cu contamination is mostly distributed in loam and clay fractions, due to their high contents in carbonates, organic matter and clay minerals, which interact with Cu. It demonstrates that organic acid washing coupled with sonication, particularly at high frequency US, may lead to significant Cu extraction yields.

Washing with acidic media leads to dissolution of carbonates and release of the Cu fixed in them. Citric acid allows to enhance Cu release by complexation of copper ionic species, in addition to dissolution. When washing is performed with a solution of humic acid sodium salt, the depollution is slower and extraction yields are lower. Depollution is here attributed to ion exchange and entrapments of Cu-containing particles in micelles formed by humic species.

These plural mechanisms (fast initial dissolution followed by a slower diffusion-controlled reaction) were modelled by a parabolic equation. In most cases, a clear increase in k_diff_ was observed in the presence of US, especially at high frequency, in agreement with the well-known accelerated diffusion under US. At low US frequency, this beneficial effect was counterbalanced by readsorption on sites created by fragmentation or disaggregation. Sonication increased the kinetics but did not affect the final extraction yields. Interestingly, the impact of US appeared to strongly depend on the soil size fraction. With citric acid, it was particularly marked on fine loam but inexistent on fine sand, which was attributed to the desorbed Cu in sand being bound to carbonates, whose dissolution was already very fast in silent conditions. On the contrary, in NaH solution, decontamination was strongly enhanced by US in fine sand, much less in fine loam. This different trend was explained by the entrapment of Cu-containing particles in micelles. Loam being finer than sand, it may be already small enough. As for sand particles, they get broken or deagglomerated by US, yielding some smaller particles. These findings highlight the need to properly characterize the soil to understand decontamination and the effect of US.

## CRediT authorship contribution statement

**Rita Salameh:** Writing – original draft, Investigation, Data curation. **Antoine Leybros:** Writing – review & editing, Writing – original draft, Validation, Supervision, Project administration, Methodology, Funding acquisition, Formal analysis, Conceptualization. **Stéphanie Szenknect:** Writing – review & editing, Validation, Methodology. **Rachel Pflieger:** Writing – review & editing, Writing – original draft, Validation, Supervision, Project administration, Methodology, Funding acquisition, Formal analysis, Conceptualization.

## Declaration of competing interest

The authors declare that they have no known competing financial interests or personal relationships that could have appeared to influence the work reported in this paper.

## Appendix A Supplementary data

Supplementary Data 1 Supplementary data: Evolution of the conductivity of the solution contacting the soil; optimisation of humic acid concentration and pH; granulometric analysis of the soil constituents; SEM images of the soil; Cu(II) speciation diagrams; TGA-MS analysis of the different soil fractions; mass of the contaminated soil fractions and corresponding copper contents; summary of obtained Cu removal yields and initial decontamination rates; estimation of the dissolved humic acid concentration via Total Organic Carbon measurements.
