# Supplementary material for: Remediation of sandy loam soil contaminated with copper by washing assisted with ultrasound
Source: Ultrason Sonochem. 2026 May 17;130:107893. doi: 10.1016/j.ultsonch.2026.107893 (PMC13214535; doi:10.1016/j.ultsonch.2026.107893)
Supplement: Supplementary Data 1 — Supplementary data: Evolution of the conductivity of the solution contacting the soil; optimisation of humic acid concentration and pH; granulometric analysis of the soil constituents; SEM images of the soil; Cu(II) speciation diagrams; TGA-MS analysis of the different soil fractions; mass of the contaminated soil fractions and corresponding copper contents; summary of obtained Cu removal yields and initial decontamination rates; estimation of the dissolved humic acid concentration via Total Organic Carbon measurements. [file mmc1.docx]

**Supporting Information**

**Remediation of sandy loam soil contaminated with copper by washing assisted with ultrasound**

**Rita Salameh^1-2^, Antoine Leybros^2^, Stéphanie Szenknect^1^, Rachel Pflieger^1^**

*^1^ICSM, Univ Montpellier, CEA, CNRS, ENSCM, Bagnols-sur- Cèze 30207, France*

*^2^CEA, DES, ISEC, DMRC, Univ Montpellier, Marcoule, France*


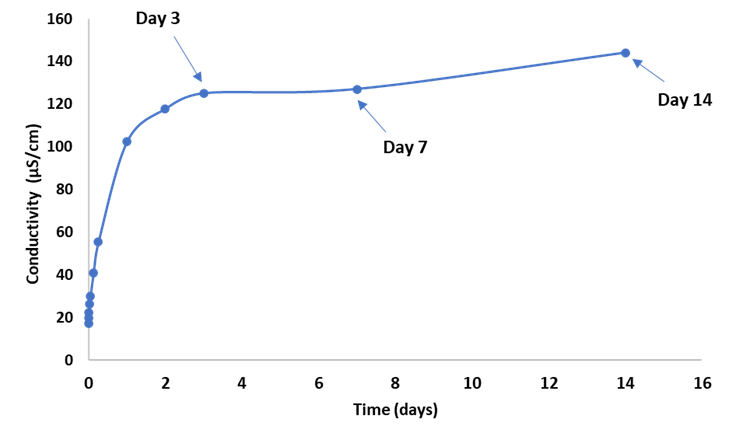


**Figure 1-SI**: Evolution of the conductivity of the solution contacting the soil over 14 days (1g of soil in contact with 100 mL of ultrapure water).

**
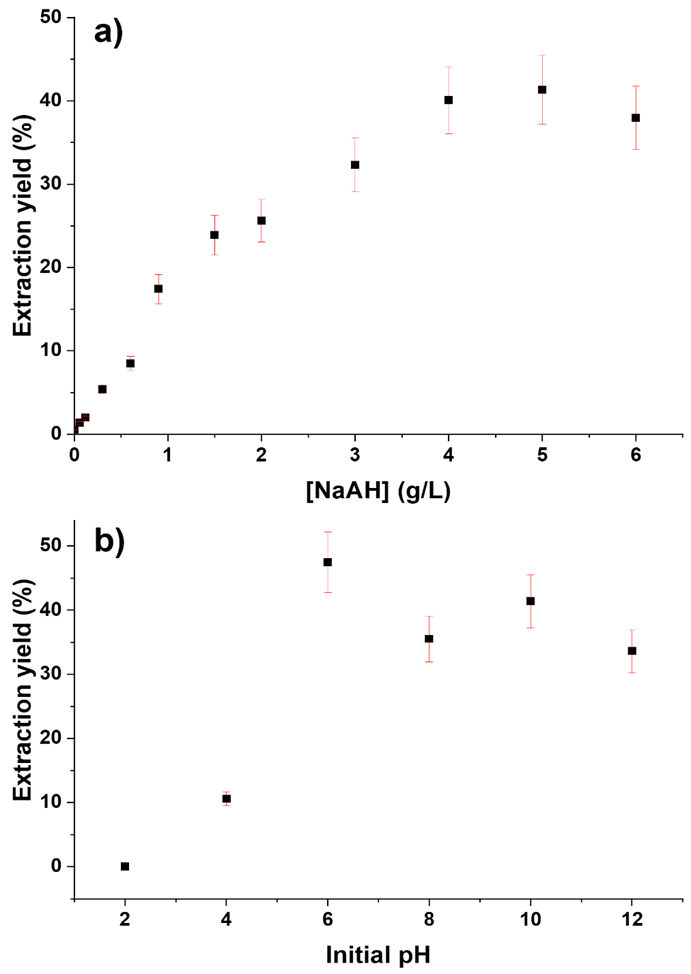
**

**Figure 2-SI**: Cu(II) extraction rate (%) a) at pH 10 and humic acid concentrations from 0 to 6 g/L b) at pH values between 2 and 12 and a concentration of 4.5 g/L of humic acid.

**
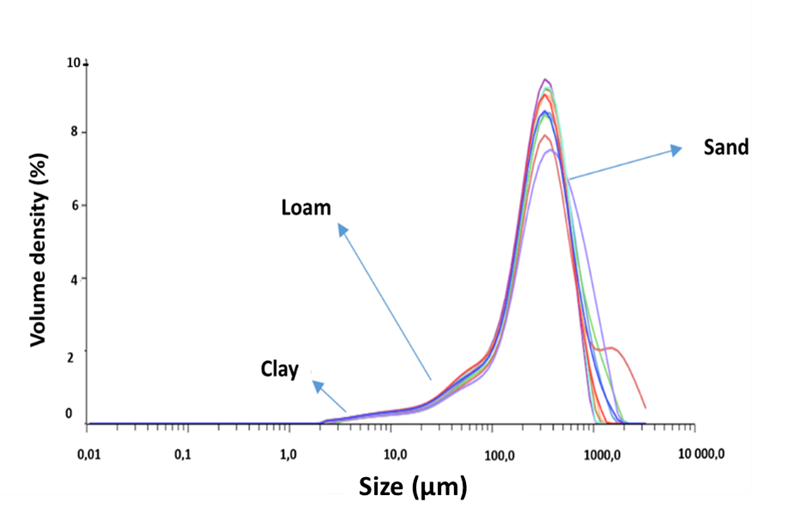
**

**Figure 3-SI**: Granulometric analysis of the soil constituents.

**
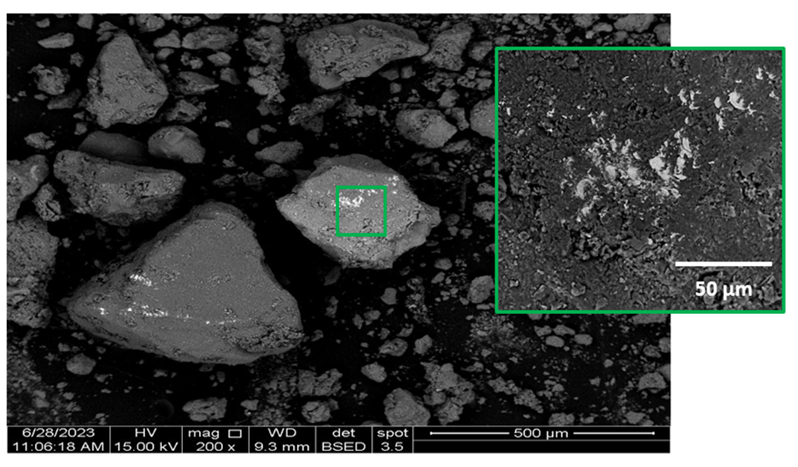
**

**Figure 4-SI**: SEM images of the soil sample in its initial state.


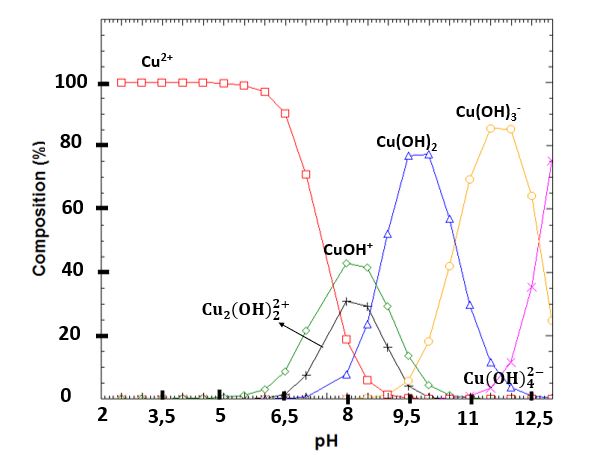


***Figure 5-SI****: Speciation diagrams of Cu(II) calculated using PhreeqC for a solution containing 1.54.10^-4^ M Cu(II) at different pH values.*


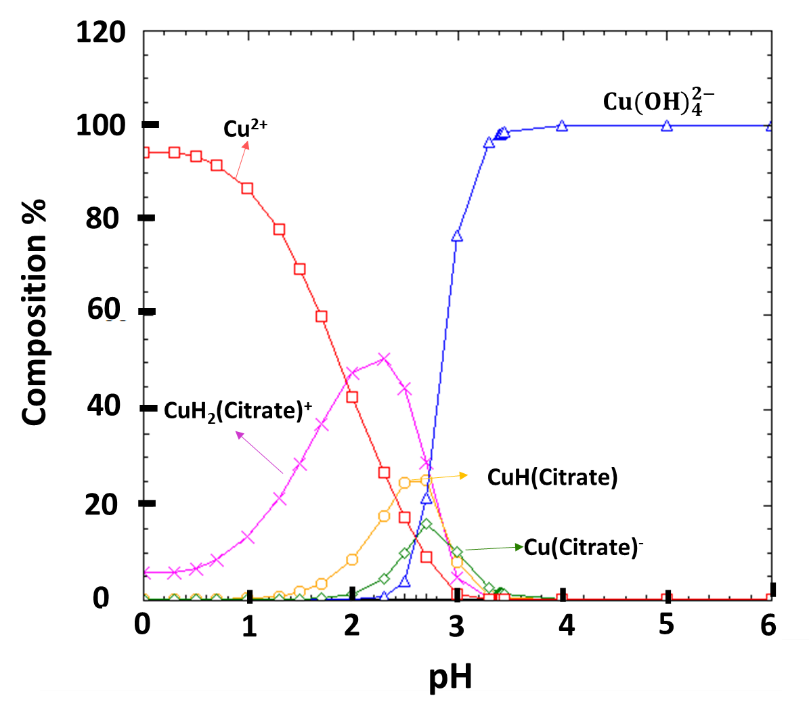


**Figure 6-SI**: Speciation diagrams of Cu(II) calculated using PhreeqC for a solution containing 1.54.10^-4^ M Cu(II) and 0.2 M citrate.

**Table 1-SI**: TGA-MS analysis of the different soil fractions (unfortunate dysfunctioning of the MS during the fine loam analysis)

| **Soil fraction** | **Time evolution of sample temperature, mass and differential mass loss** | **MS intensities of H_2_O and CO_2_ vs. time** |
| --- | --- | --- |
| **Unseparated soil** | 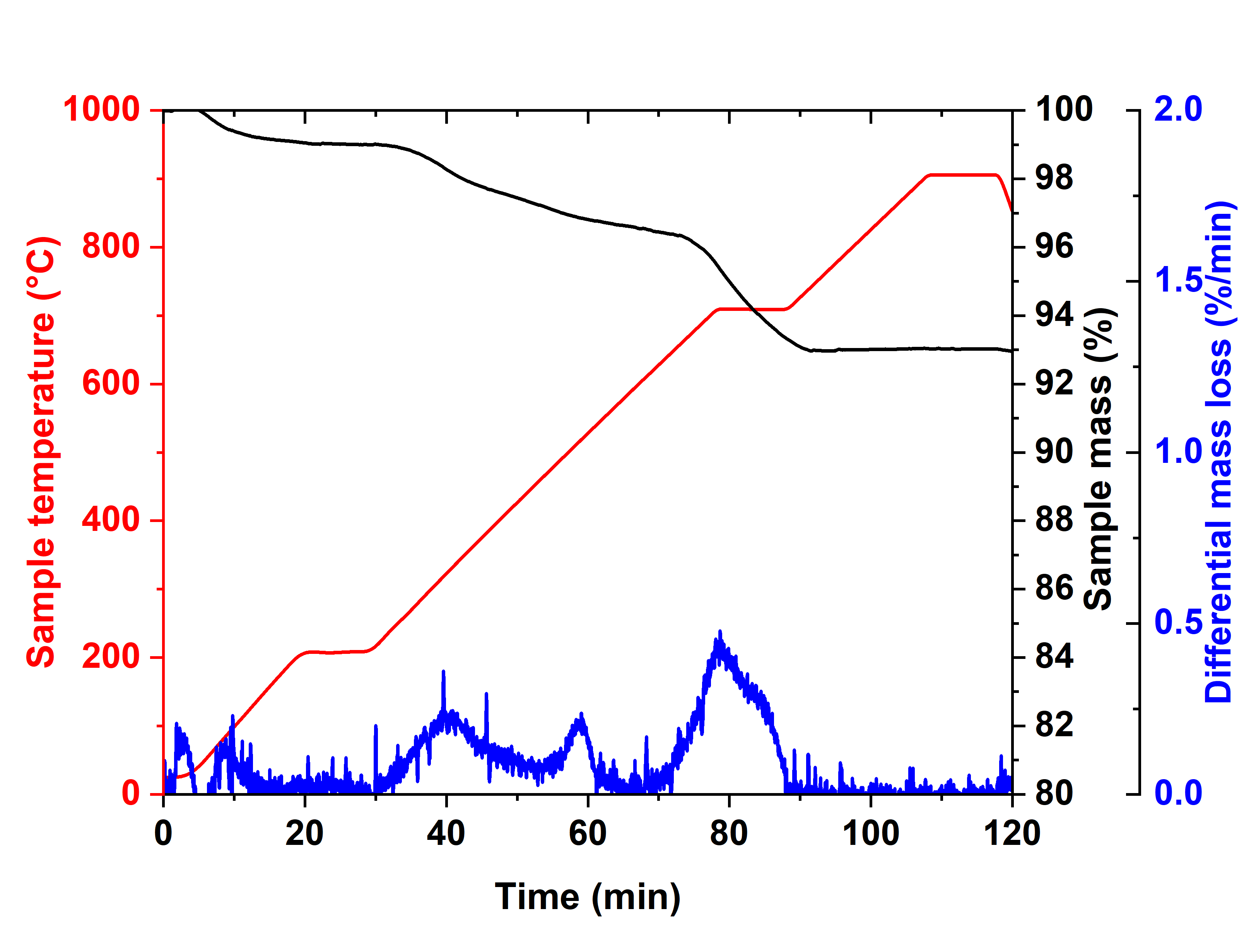 | 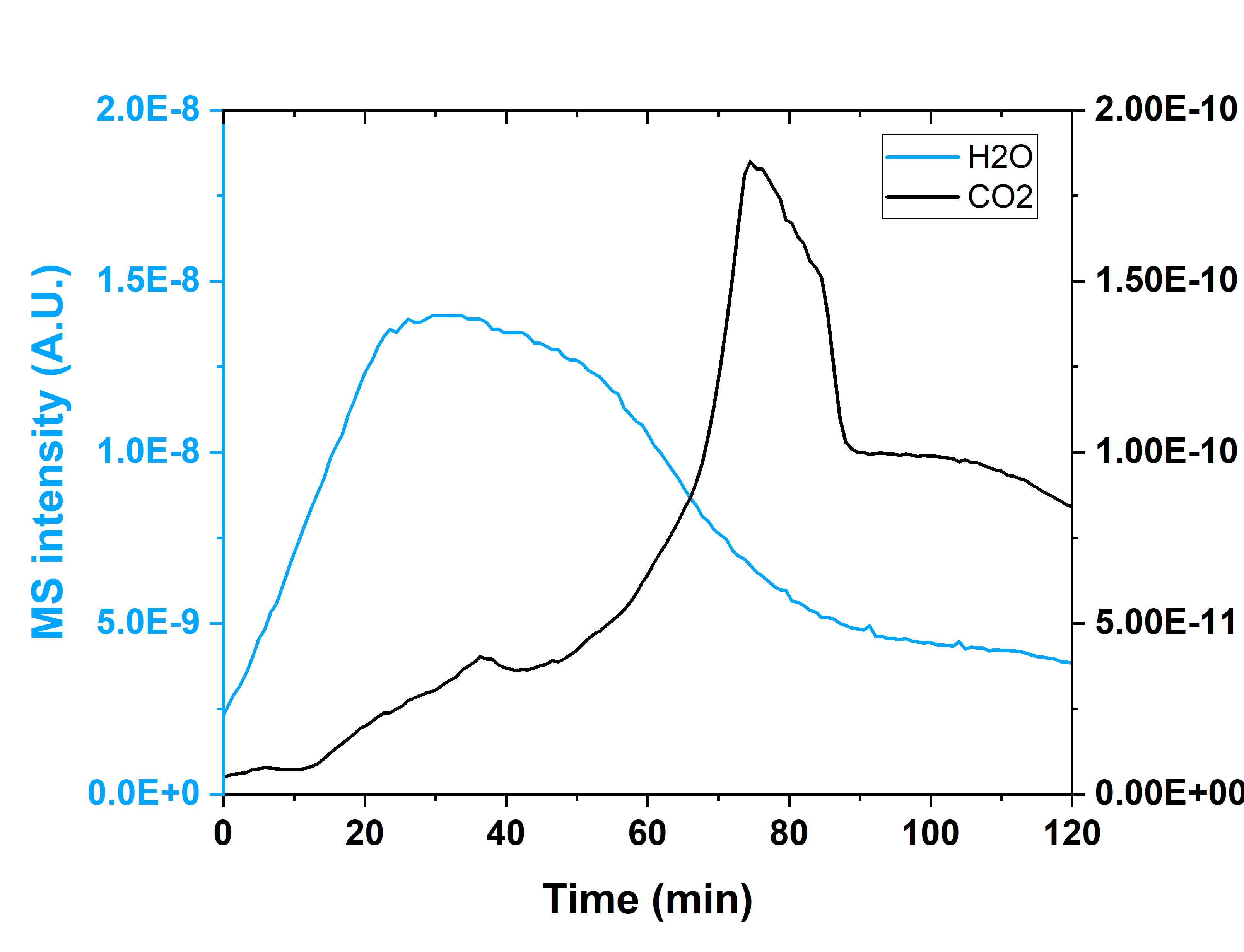 |
| **Coarse sand** | 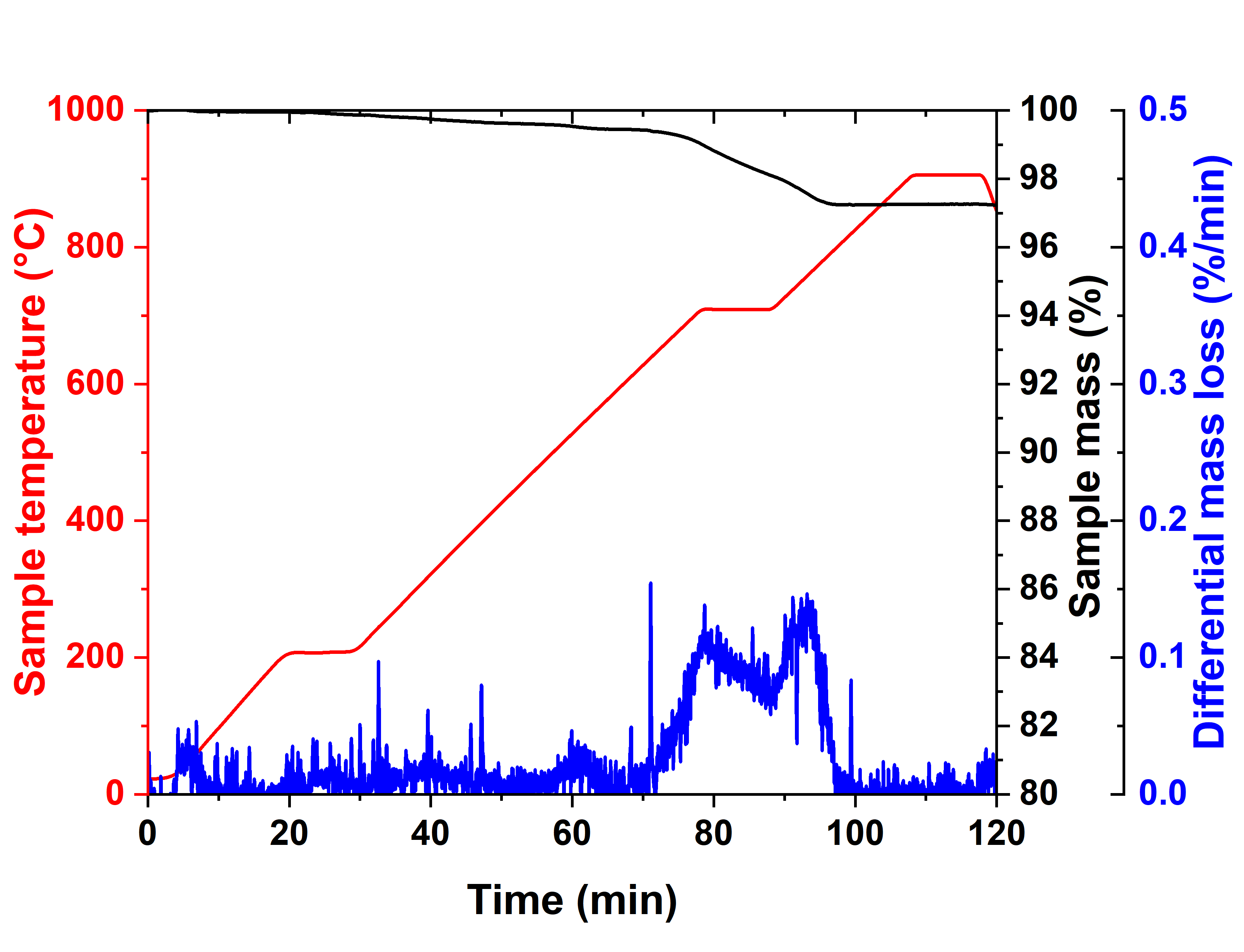 | 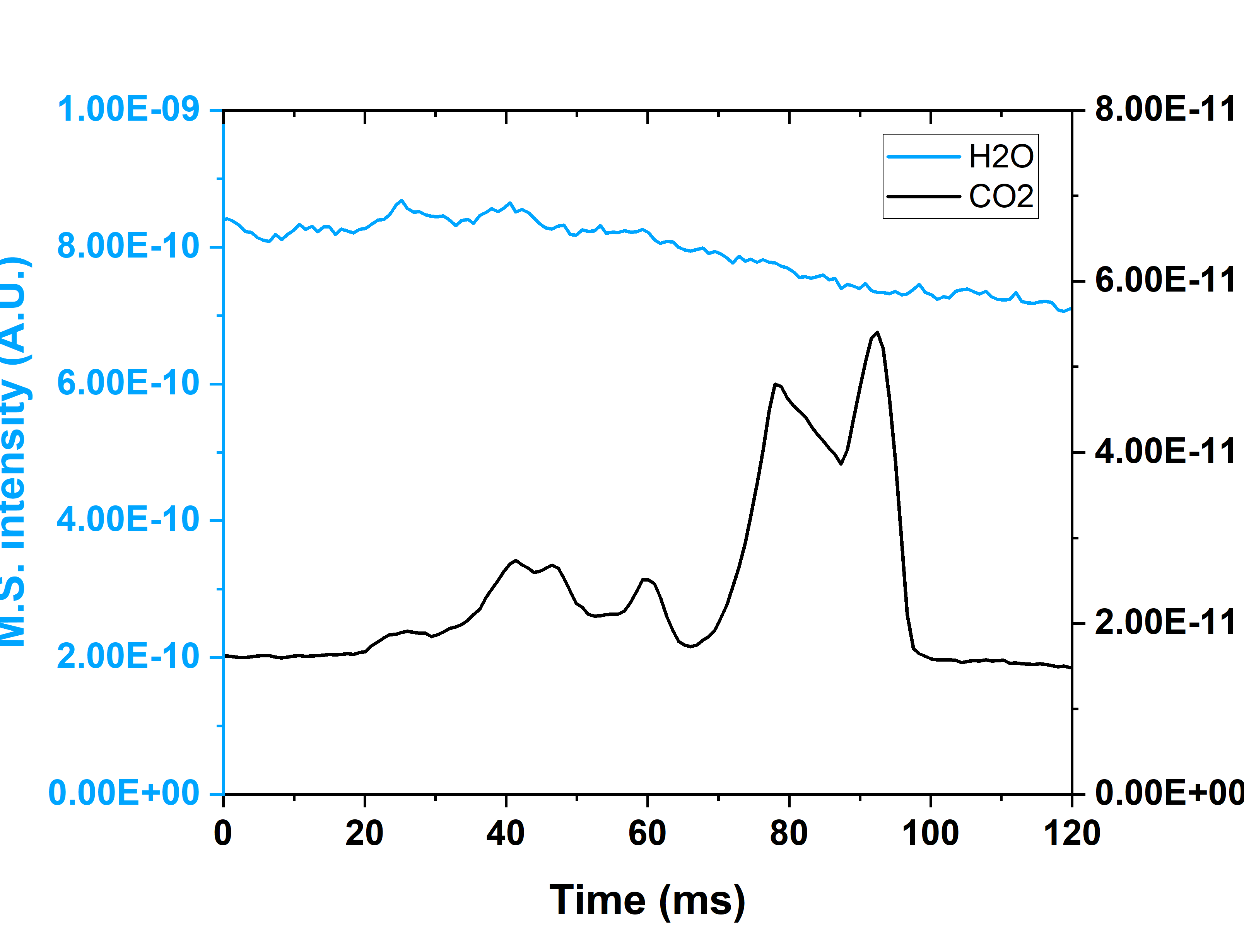 |
| **Fine sand** | 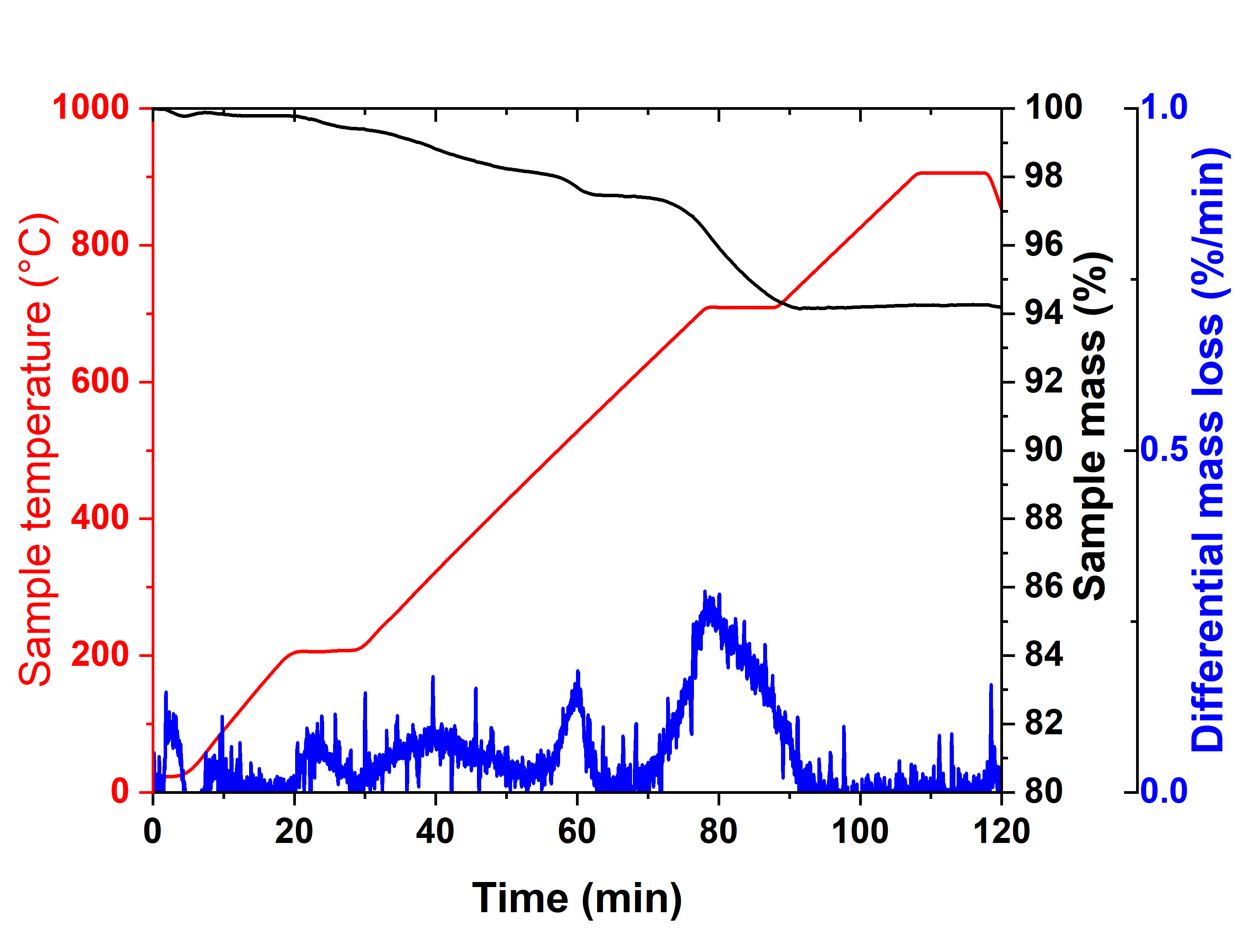 | 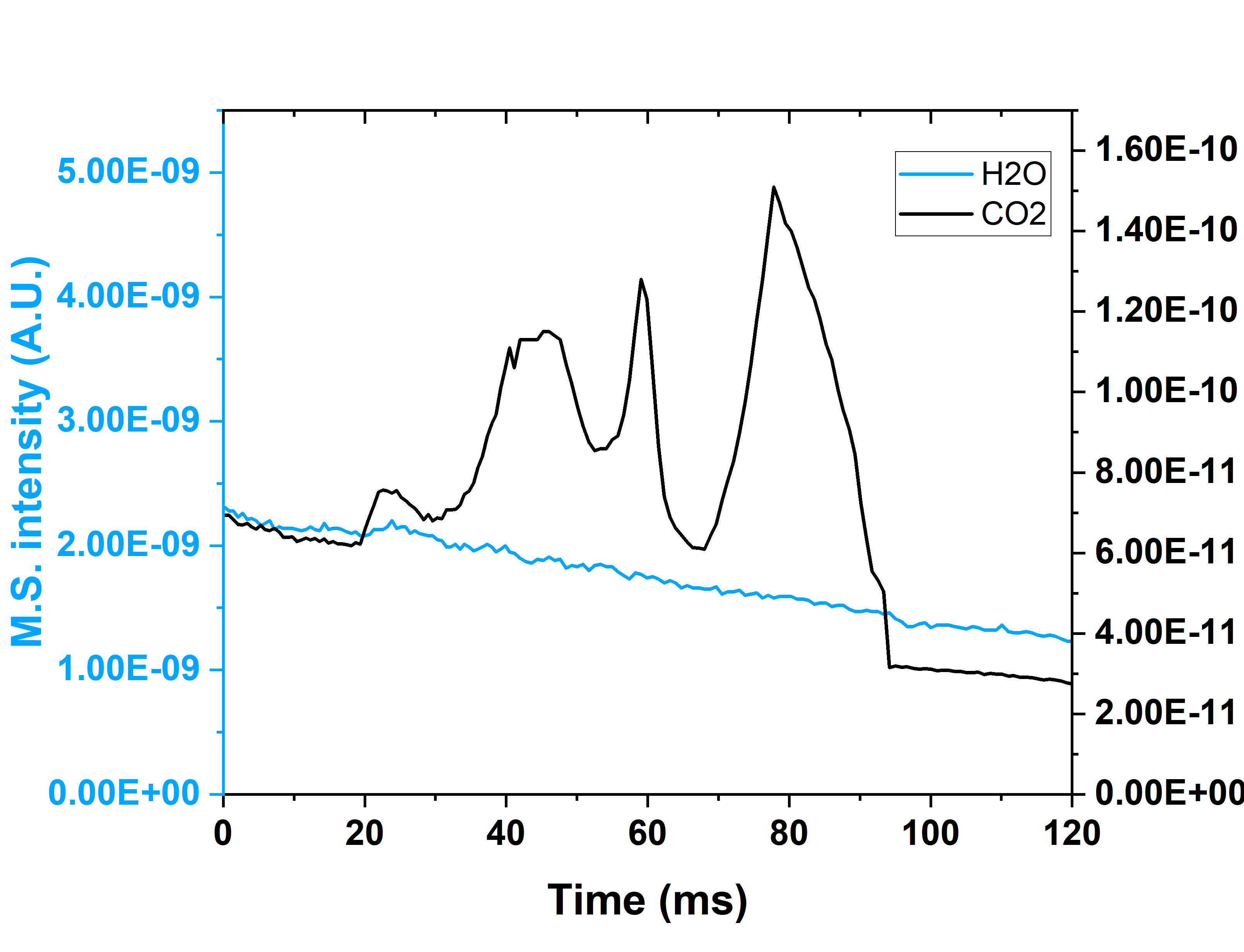 |
| **Coarse loam** | 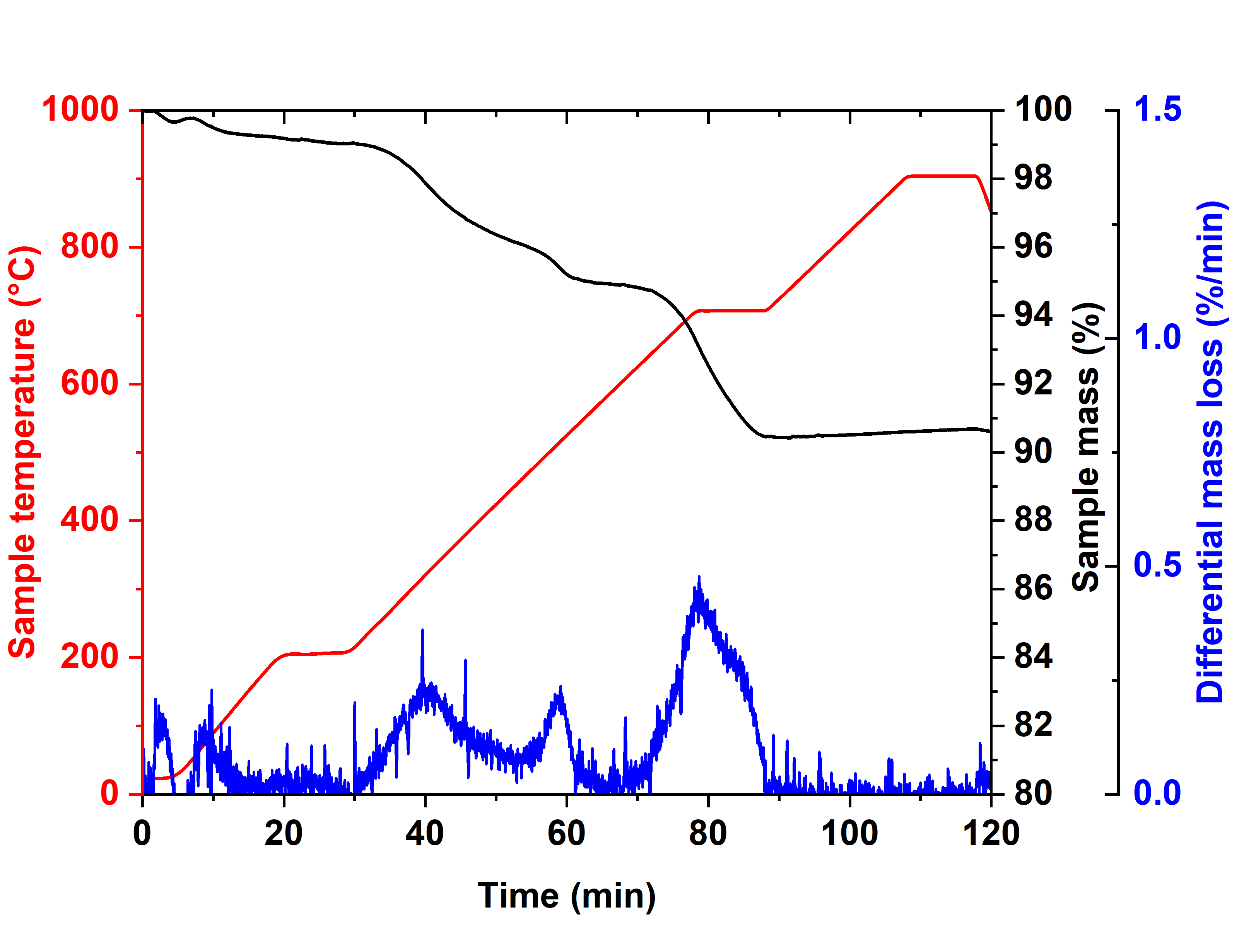 | 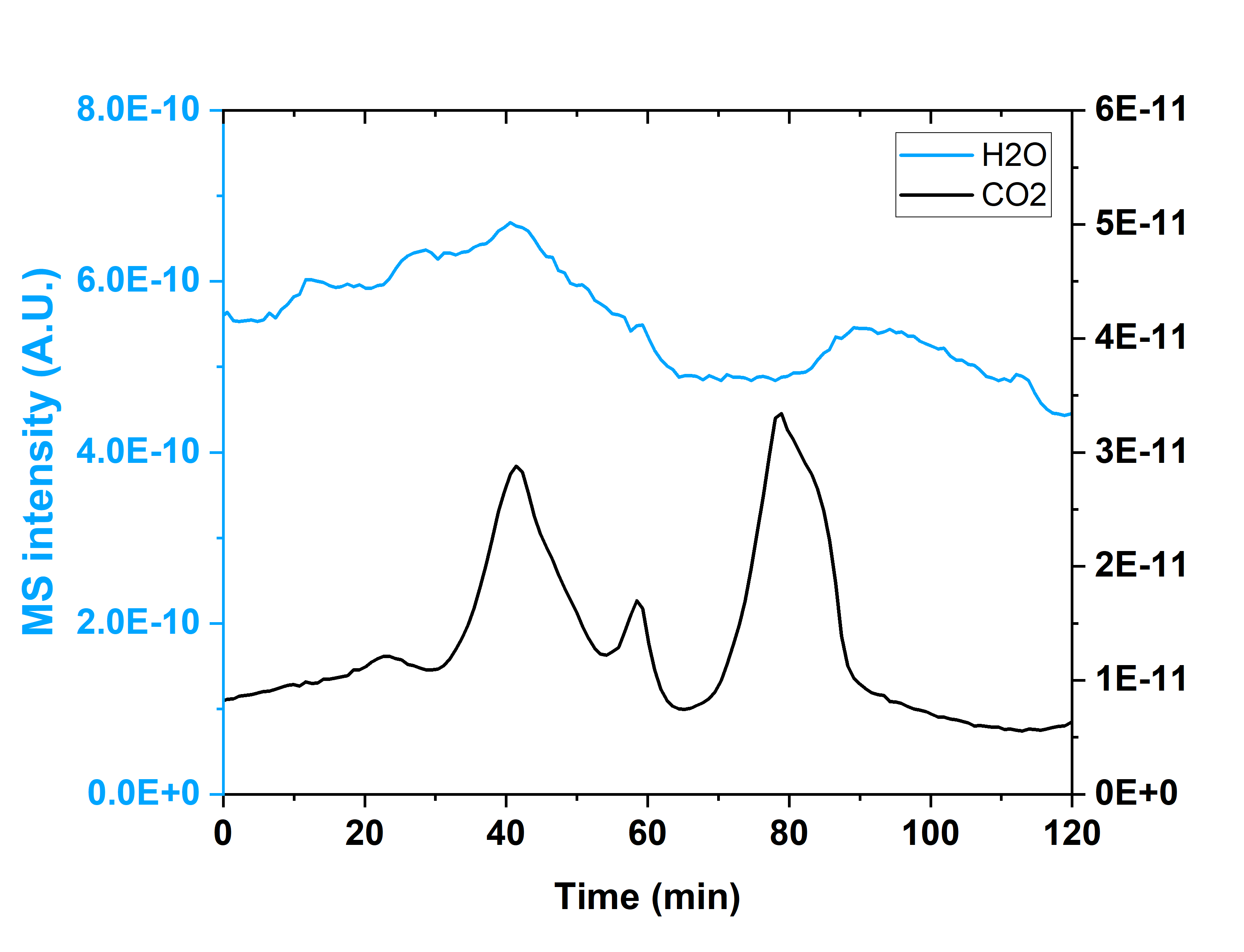 |
| **Fine loam** | 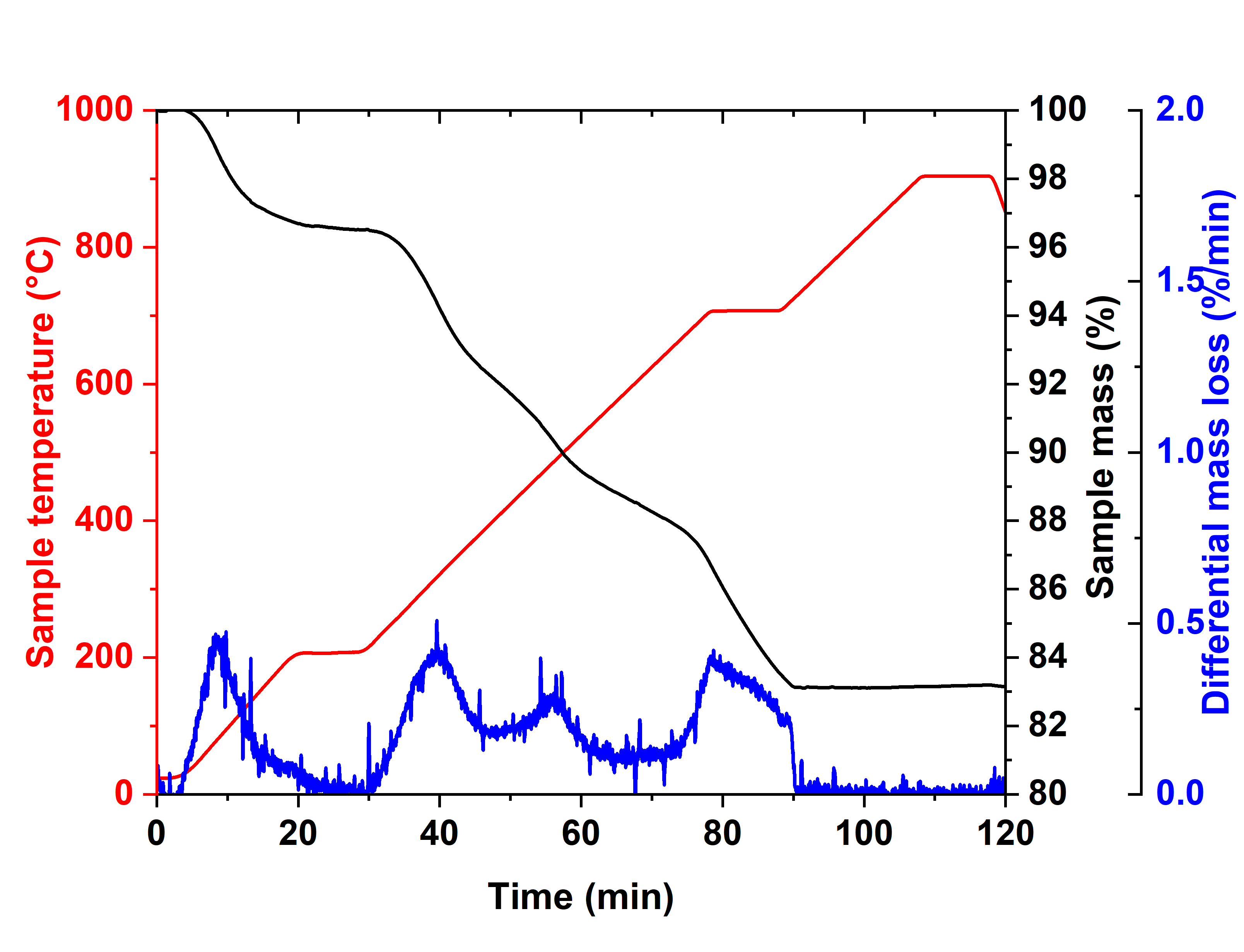 | - |
| **Clay** | 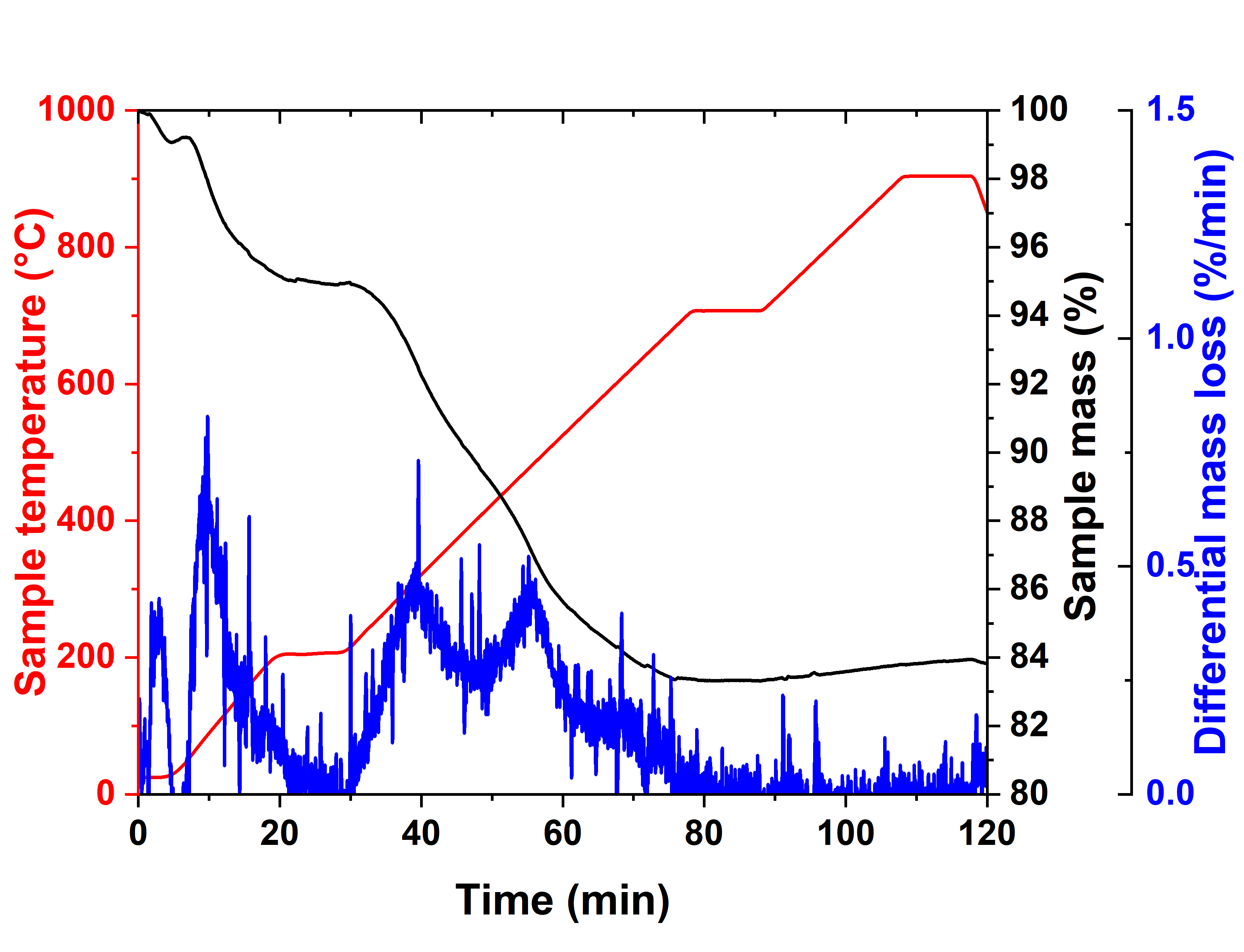 | 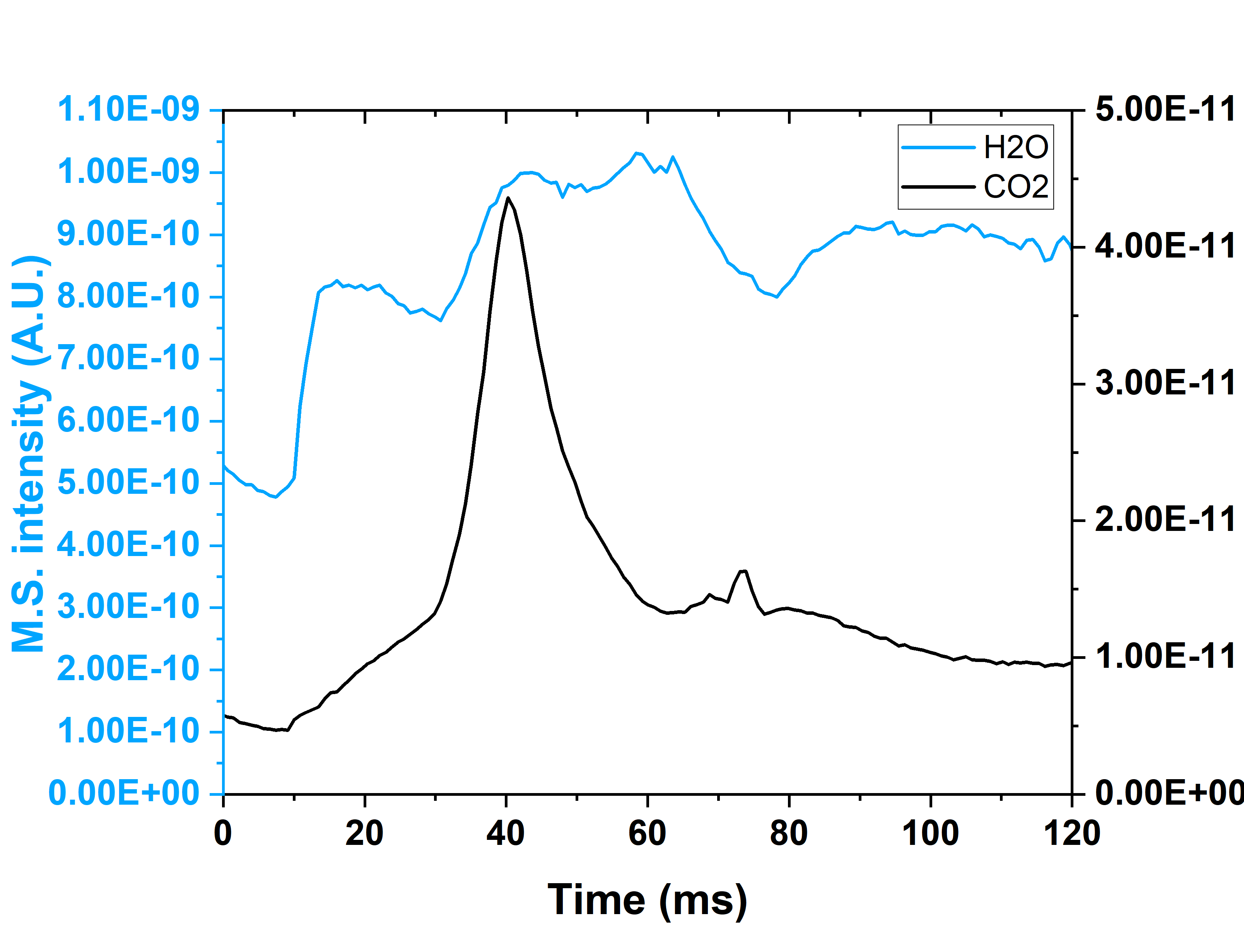 |

***Table 2-SI:*** *Mass of the contaminated soil fractions (in g) and corresponding copper contents (in mg and mg/g).*

|  | Coarse sand | Fine sand | Coarse silt | Fine silt | Clay | Uncertainty |
| --- | --- | --- | --- | --- | --- | --- |
| mg of Cu/ g of fraction  (mg/g) | 0.01 | 0.02 | 0.16 | 0.36 | 0.5 | ± 10% |
| mass of fraction (g) | 6.30 | 2.97 | 0.85 | 1.74 | 0.26 | ± 11% |
| mass of total copper in each fraction (mg) | 0.06 | 0.06 | 0.14 | 0.63 | 0.15 | ± 21% |

**Table 3-SI:** Summary showing the standard error**,** correlation coefficient, diffusion constant obtained using parabolic kinetic model and decontamination speed for 3 different samples using 2 washing solutions citric acid and sodium salt humic acid under 3 experimental conditions silent, ultrasonic bath 45 kHz and high frequency 358 kHz.

2

|  | |  |  |  | **Experimental conditions** | | | | | | | | | | | |  |
| --- | --- | --- | --- | --- | --- | --- | --- | --- | --- | --- | --- | --- | --- | --- | --- | --- | --- |
|  |  | **Silent** | **45 kHz** | **358 kHz** | **Silent** | **45 kHz** | **358 kHz** | **Silent** | **45 kHz** | **358 kHz** | **Silent** | **45 kHz** | **358 kHz** | **Silent** | **US bath 45 kHz** | **358 kHz** |  |
| **Solutions** | **Samples** | **1-h yields ± 10% (%)** | | | **V_0_ ± 14% (extraction % per minute)** | | | **k_diff_ (diffusion constant) (mg.kg^-1^.s^-0.5^)** | | | **SE (standard deviation)** | | | **R^2^ (coefficient of correlation)** | | |  |
| NaH | Unseparated soil | 23.6 | 30.5 | 37.6 | 4.8 | 6.8 | 7.1 | 3.4 **±** 0.10 | 4.1 **±** 0.16 | 3.7 **±** 0.07 | 0.31 | 0.56 | 0.35 | 0.99 | 0.98 | 0.99 |  |
|  |  |  |  |  |  |  |  |  |  |  |  |  |  |  |  |  |  |
|  |  |  |  |  |  |  |  |  |  |  |  |  |  |  |  |  |  |
|  |  |  |  |  |  |  |  |  |  |  |  |  |  |  |  |  |  |
| AC | Unseparated soil | 61.9 | 65.4 | 65.5 | 25.9 | 25.5 | 24.8 | 1.2 ± 0.08 | 2.6 **±** 0.13 | 3.3 **±** 0.28 | 0.29 | 0.48 | 0.94 | 0.97 | 0.97 | 0.93 |  |
|  |  |  |  |  |  |  |  |  |  |  |  |  |  |  |  |  |  |
|  |  |  |  |  |  |  |  |  |  |  |  |  |  |  |  |  |  |
| NaH | Fine loam | 24.4 | 26.3 | 26.7 | 4.5 | 6.4 | 5.6 | 8.6 **±** 0.08 | 8.6 **±** 0.18 | 10.7 **±** 0.31 | 0.24 | 0.55 | 0.79 | 0.99 | 0.97 | 0.95 |  |
|  |  |  |  |  |  |  |  |  |  |  |  |  |  |  |  |  |  |
|  |  |  |  |  |  |  |  |  |  |  |  |  |  |  |  |  |  |
| AC | Fine loam | 62.0 | 65.6 | 85.8 | 25.8 | 26 | 32.2 | 5.1 ± 0.38 | 5.7 ± 0.09 | 7.9 ± 0.07 | 0.38 | 0.62 | 0.36 | 0.96 | 0.96 | 0.99 |  |
|  |  |  |  |  |  |  |  |  |  |  |  |  |  |  |  |  |  |
|  |  |  |  |  |  |  |  |  |  |  |  |  |  |  |  |  |  |
| NaH | Fine sand | 18.1 | 37.5 | 43.2 | 0.01 | 0.5 | 0.7 | 1,0 **±** 0.06 | 1.5 **±** 0.27 | 1,0 **±** 0.20 | 0.39 | 0.76 | 0.9 | 0.99 | 0.97 | 0.99 |  |
|  |  |  |  |  |  |  |  |  |  |  |  |  |  |  |  |  |  |
|  |  |  |  |  |  |  |  |  |  |  |  |  |  |  |  |  |  |
| AC | Fine sand | 36.4 | 35.4 | 36.2 | 13.1 | 14.2 | 13.7 | 0.9 **±** 0.22 | 0.9 **±** 0.22 | 1.1 ± 0.20 | 0.52 | 0.3 | 0.45 | 0.94 | 0.92 | 0.91 |  |
|  |  |  |  |  |  |  |  |  |  |  |  |  |  |  |  |  |  |
|  |  |  |  |  |  |  |  |  |  |  |  |  |  |  |  |  |  |

***Table 4-SI:*** *Estimation of the dissolved humic acid concentration via Total Organic Carbon measurements (mg/L) after filtration of NaH suspensions in water at different pH values (± 10 %).*

| **pH** | 4 | 6 | 8 | 10 | 12 |
| --- | --- | --- | --- | --- | --- |
| **TOC (mg/L)** | 3.22 | 3.42 | 3.67 | 3.66 | 3.94 |
